# Aromatic Constituents from the Leaves of *Actinidia arguta* with Antioxidant and α-Glucosidase Inhibitory Activity

**DOI:** 10.3390/antiox10121896

**Published:** 2021-11-26

**Authors:** Jong Hoon Ahn, Se Hwan Ryu, Solip Lee, Sang Won Yeon, Ayman Turk, Yoo Kyong Han, Ki Yong Lee, Bang Yeon Hwang, Mi Kyeong Lee

**Affiliations:** 1 College of Pharmacy, Chungbuk National University, Cheongju 28160, Korea; jhahn@dodamip.com (J.H.A.); sehwan0188@chungbuk.ac.kr (S.H.R.); dudaos00@chungbuk.ac.kr (S.L.); sangwon1352@chungbuk.ac.kr (S.W.Y.); aymanturk@chungbuk.ac.kr (A.T.); byhwang@chungbuk.ac.kr (B.Y.H.); 2 College of Pharmacy, Korea University, Sejong 47236, Korea; yookyong05@korea.ac.kr (Y.K.H.); kylee11@korea.ac.kr (K.Y.L.)

**Keywords:** *Actinidia arguta*, aromatic, argutosides A–E, antioxidant, α-glucosidase, molecular docking analysis

## Abstract

As the leaf of *Actinidia arguta* has shown antioxidant activity, a study was conducted to identify the active ingredients. Forty-eight compounds were isolated from the leaves of *A. arguta* through various chromatographic techniques. Further characterization of the structures on the basis of 1D and 2D NMR and MS data identified several aromatic compounds, including phenylpropanoid derivatives, phenolics, coumarins, flavonoids and lignans. Among them, five compounds were newly reported, naturally occurring, and named argutosides A–D (**1**–**4**), which consist of phenylpropanoid glycosides that are conjugated with a phenolic moiety, and argutoside E (**5**), which is a coumarin glycoside that is conjugated with a phenylpropanoid unit. The isolated compounds showed good antioxidant and α-glucosidase inhibitory activity with differences in activity depending on the structures. Molecular docking analysis demonstrated the interaction between the hydroxyl and carbonyl groups of compounds **1** and **5** with α-glucosidase. Taken together, the leaves of *A. arguta* are rich in aromatic compounds with diverse structures. Therefore, the leaves of *A. arguta* and their aromatic components might be beneficial for oxidative stress and glucose-related diseases.

## 1. Introduction

Oxidative stress is caused by the excessive production of reactive oxygen species (ROS) which bind to molecules in vivo and consequently alter their structures and functions. An antioxidant-related defense mechanism exists to protect against generated ROS. However, persistent oxidative stress by excessive production of ROS eventually leads to diverse severe diseases such as cancer, inflammation and metabolic diseases [1,2,3].

Diabetes is a metabolic disease with a high incidence worldwide. In diabetes, the blood glucose level increases due to the abnormal operation of insulin, which causes various complications and develops into a serious disease [4]. Various factors are known to be involved in the onset and progression of diabetes; oxidative stress is one such mediator [5,6]. The increased ROS attack the pancreas and interfere with the normal function of insulin [7,8]. In other words, oxidative stress and diabetes are mutually detrimental to each other [9,10].

Accordingly, research into the development of a therapeutic agent for diabetes is being actively conducted. α-Glucosidase is an intestinal enzyme which converts carbohydrates into single monosaccharides. Therefore, α-glucosidase inhibitors are used for the treatment of diabetes and carbohydrate-mediated diseases [11,12]. Antioxidants are also used for the prevention and treatment of diabetes. 

Natural products are good sources for antioxidants and are widely used in the prevention and treatment of various diseases [13]. In particular, polyphenols are representative components with antioxidant action and are present in various plants. In addition, they also have therapeutic potential for metabolic diseases and have shown excellent results in various diabetes models [14,15]. 

*Actinidia arguta*, also called hardy kiwifruit or kiwiberry, has small fruits with a smooth green skin. Due to its cold-resistant characteristic, it can be cultivated in Northeast Asia [16,17]. The grains of *A. arguta* are mainly consumed as fresh fruits while the cooked leaves are used in the treatment of various diseases with antioxidant, antibacterial, antidiabetic and anti-inflammatory effects [18,19,20]. We recognized the importance of *A. arguta* as a native plant together with its biological activities and, thus, investigated the efficacy and ingredients of *A. arguta*. As a follow-up study on *A. arguta* leaves [21], the antioxidant and anti-diabetic effects of the extracts were confirmed. Further investigation into the bioactive constituents of *A. arguta* leaves resulted in the isolation of 48 compounds, including five new compounds. On the basis of 1D and 2D NMR and MS data, the structures of the isolated compounds were determined to be aromatic and included phenylpropanoid derivatives, phenolics, coumarins, flavonoids and lignans. The antioxidant and α-glucosidase inhibitory activity of the isolated compounds were measured and their mechanism of action was analyzed using molecular docking analysis. 

## 2. Materials and Methods

### 2.1. Plant Material

The leaves of *A. arguta* were obtained from a farm in Gwangyang, South Korea (GPS: DD 34.990714, 127.591508) in August 2016. After identification by the herbarium of the College of Pharmacy Chungbuk National University, voucher specimens (CBNU2016-AAL) were deposited in a specimen room of the herbarium.

### 2.2. General Experimental Procedure

The UV and IR spectra were obtained using Jasco UV-550 (JASCO, Tokyo, Japan) and Perkin–Elmer model LE599 (Perkin–Elmer, Waltham, MA, USA) spectrometer, respectively. A Bruker DRX 400 or 500 MHz spectrometer (Bruker-Biospin, Karlsruhe, Germany) were used for the analysis of NMR signals using CD_3_OD as a solvent. ESIMS and HRESI-TOF-MS data were obtained on LCQ Fleet and maXis 4G mass spectrometers (Bruker Daltonics, Bremen, Germany), respectively. Semi-preparative HPLC (Waters, Milford, MA, USA) was performed using a Waters 515 HPLC pump with a 996-photodiode array detector, and Waters Empower software using a Gemini-NX ODS-column (150 × 10.0 mm and 150 × 21.2 mm). Column chromatography procedures were performed using silica gel (200–400 mesh, Fisher Scientific, Waltham, MA, USA) and Sephadex LH-20 (25–100 µm, Pharmacia Fine Chemical Industries Co., Uppsala, Sweden). Thin-layer chromatography (TLC) was performed using aluminum plates precoated with Kieselgel 60 F_254_ (0.25 mm, Merck, Darmstadt, Germany). 

### 2.3. Extraction and Isolation

The dried powder of *A. arguta* leaves (4.0 kg) was extracted with 80% MeOH (30 L × 2) at room temperature. The MeOH extract (350.0 g) was suspended in H_2_O (2 L) and partitioned successively with *n*-hexane, CH_2_Cl_2,_ EtOAc and *n*-BuOH (each 2L × 2) for 24 h.

The CH_2_Cl_2_ fraction (AALC, 24.1 g) was chromatographed on silica gel and eluted with a mixture of *n*-hexane-EtOAc (100% *n*-hexane to 100% EtOAc) to obtain fourteen subfractions (AALC1-C14). Subfraction C13 (3.2 g) was subjected to MPLC on RP-silica gel and eluted with mixtures of MeOH-H_2_O (5% MeOH to 100% MeOH) to give three subfractions (C13A-C13C). Compounds **12** and **43** were purified from C13B and C13C, respectively, by semi-preparative HPLC and eluted with acetonitrile-H_2_O (20:80). Compounds **41**, **42** and **47** were purified from C14 F by Sephadex LH-20 and eluted with MeOH followed by semi-preparative HPLC and elution with acetonitrile-H_2_O (20:80). 

The EtOAc fraction (AALE, 24.4 g) was chromatographed on silica gel and eluted with a mixture of CH_2_Cl_2_-MeOH by step gradient (100% CH_2_Cl_2_ to 100% MeOH) to obtain eleven subfractions (AALE1-E11). Subfraction E4 (2.5 g) was subjected to MPLC on RP-silica gel and eluted with mixtures of MeOH-H_2_O (5% to 100% MeOH) to give six subfractions (E4A–E4F). Subfraction E4C was separated into two subfractions (E4F1–E4F2) by Sephadex LH-20 (MeOH). Compounds **9**, **10**, **13**, **14** and **20** were purified from E4C2 by semi-preparative HPLC (acetonitrile-H_2_O, 20:80). E5 (2.9 g) was subjected to MPLC on RP-silica gel (mixtures of MeOH-H_2_O, 5% to 100% MeOH) to give eight subfractions (E5A–E5H). Sephadex LH-20 column chromatography (MeOH) of E5B, E5C, E5G and E5H gave compounds **11**, **8**, **31** and **22**, respectively. Compounds **17** and **18** were isolated from E5D by Sephadex LH-20 (MeOH) followed by semi-preparative HPLC and elution with acetonitrile-H_2_O (30:70). Subfraction E5F was separated by Sephadex LH-20 (MeOH) to obtain E5F1 and E5F2, which gives compounds **32** and **29**, respectively, by semi-preparative HPLC and elution with acetonitrile-H_2_O (30:70). 

Subfraction E6 (3.2 g) was subjected to MPLC on RP-silica gel and eluted with mixtures of MeOH-H_2_O (5% MeOH to 100% MeOH) to give eight subfractions (E6A–E6H). Compounds **12** and **15** were purified from E6B by semi-preparative HPLC (acetonitrile-H_2_O, 50:50). Compounds **16** and **19** were purified from E6C by Sephadex LH-20 (MeOH) followed by semi-preparative HPLC (acetonitrile-H_2_O, 50:50). Compounds **30** and **25** were purified from E6D and E6F, respectively, by Sephadex LH-20 (MeOH). E7 (2.6 g) was subjected to MPLC on RP-silica gel and eluted with mixtures of MeOH-H_2_O (10% MeOH to 100% MeOH) to give nine subfractions (E7A–E7I). E7D was separated to obtain E7D1 by Sephadex LH-20 and eluted with MeOH. Compound **40** was purified from E7D1 by semi-preparative HPLC and eluted with acetonitrile-H_2_O (30:70).

Subfraction E8 (4.2 g) was subjected to MPLC on RP-silica gel and eluted with mixtures of MeOH-H_2_O (5% to 100% MeOH) to give nine subfractions (E8A–E8I). E8C, E8D and E8E were subjected to Sephadex LH-20 (MeOH) to give compound **37, 36** and **38**, respectively. Subfraction E8G was separated by Sephadex LH-20 (MeOH) to obtain E8G1 and E8G2, which gives compounds **44** and **39**, respectively, by semi-preparative HPLC and elution with MeCN-H2O (20:80). E8I (2.9 g) was subjected to Sephadex LH-20 (MeOH) to give four subfractions (E8I1–E8I4). Compounds **45** and **46** were isolated from E8I2 by semi-preparative HPLC (acetonitrile-H_2_O, 18:82). Semi-preparative HPLC (acetonitrile-H_2_O, 18:82) of E8I4 gives compounds **1**, **5**, **6**, **7** and **28**.

Subfraction E9 (5.0 g) was subjected to MPLC on RP-silica gel and eluted with mixtures of MeOH-H_2_O (5% to 100% MeOH) to give ten subfractions (E9A–E9J). Compounds **33, 23, 34** and **35** were isolated from E9E, E9H, E9I and E9J, respectively, by Sephadex LH-20 (MeOH). Subfraction E9H was subjected to Sephadex LH-20 (MeOH) to give 3 subfractions (E9H1–E9H3). Compounds **2** and **3** were purified from E9H2 by semi-preparative HPLC (acetonitrile-H_2_O, 20:80). Subfraction E9I was subjected to Sephadex LH-20 (MeOH) to give 4 subfractions (E9I1–E9I4). Compounds **4** and **48** were obtained from E9I2 by semi-preparative HPLC (acetonitrile-H_2_O, 23:77). Compounds **24, 26** and **27** were purified from E9I3 by semi-preparative HPLC (MeCN-H_2_O, 20:80).

#### 2.3.1. Argutoside A (**1**)

Brown syrup; IR ν_max_ 3411, 1664 cm^−1^; ^1^H-NMR (400 MHz, CD_3_OD) and ^13^C-NMR (100 MHz, CD_3_OD), see Tables 1 and 2, Figures S1–S4; HRESI-TOF-MS (positive mode) m/z 511.1210 (calcd. for C_24_H_24_NaO_11_, 511.1216, Figure S5).

#### 2.3.2. Argutoside B (**2**)

Brown syrup; IR ν*_max_* 3411, 1631 cm^−1^; ^1^H-NMR (400 MHz, CD_3_OD) and ^13^C-NMR (100 MHz, CD_3_OD), see Tables 1 and 2, Figures S6–S9; HRESI-TOF-MS (positive mode) *m/z* 485.1418 (calcd. for C_23_H_26_NaO_10_, 485.1424, Figure S10). 

#### 2.3.3. Argutoside C (**3**)

Brown syrup; IR ν*_max_* 3411, 1666 cm^−1^; ^1^H-NMR (400 MHz, CD_3_OD) and ^13^C-NMR (100 MHz, CD_3_OD), see Tables 1 and 2, Figures S11–S14: ESIMS *m/z* 485 [M + Na]^+^; HRESI-TOF-MS (positive mode) *m/z* 485.1418 ([M + Na]^+^ calcd. for C_23_H_26_NaO_10_, 485.1424, Figure S15). 

#### 2.3.4. Argutoside D (**4**)

Brown syrup; IR ν*_max_* 3423, 1666 cm^−1^; ^1^H-NMR (400 MHz, CD_3_OD) and ^13^C-NMR (100 MHz, CD_3_OD), see Tables 1 and 2, Figures S16–S19; HRESI-TOF-MS *m/z* 529.1680 (calcd. for C_25_H_30_NaO_11_, 529.1686, Figure S20). 

#### 2.3.5. Argutoside E (**5**)

Brown syrup; IR ν*_max_* 3419, 1660 cm-1; ^1^H-NMR (400 MHz, DMSO-*d*_6_) and ^13^C-NMR (100 MHz, DMSO-*d*_6_), see Table 3 and Figures S21–S24; HRESI-TOF-MS *m/z* 525.1003 (calcd. for C_24_H_22_NaO_12_, 525.1009, Figure S25). 

### 2.4. Measurement of α-Glucosidase Activity

The inhibitory effect on α-glucosidase was measured using α-glucosidase (from *Saccharomyces cerevisiae* (EC 3.2.1.20) [21]. A test sample was mixed with 80 μL enzyme buffer and 10 μL α-glucosidase and incubated for 15 min at 37 °C. Then, after the addition of 10 μL *p*-nitrophenyl α-D-glucopyranoside solution for enzyme reaction, the amount of *p*-nitrophenol that was cleaved by the enzyme was determined by measuring the absorbance at 405 nm in a 96-well microplate reader. Acarbose was used as a positive control.

### 2.5. Measurement of DPPH Radical Scavenging Activity

The antioxidant activity was evaluated by measuring the free radical scavenging activity using DPPH as previously reported [20]. In brief, freshly prepared DPPH solution was mixed with the samples. The mixture was reacted at room temperature for 10 min, and the absorbance was measured at 550 nm. Ascorbic acid was used as a positive control.

### 2.6. Molecular Docking Studies

SYBYL-X 2.1.1 (Tripos Ltd., St. Louis, MO, USA) with crystal structures of N-terminal subunit (NtMGAM; PDB-ID: 2QMJ) and C-terminal subunit (CtMGAM; PDB-ID: 3TOP) of human maltase-glucoamylase (MGAM) were used, respectively, for molecular docking studies of active compounds [19].

## 3. Results

### 3.1. Structural Elucidation

**Figure 1:**
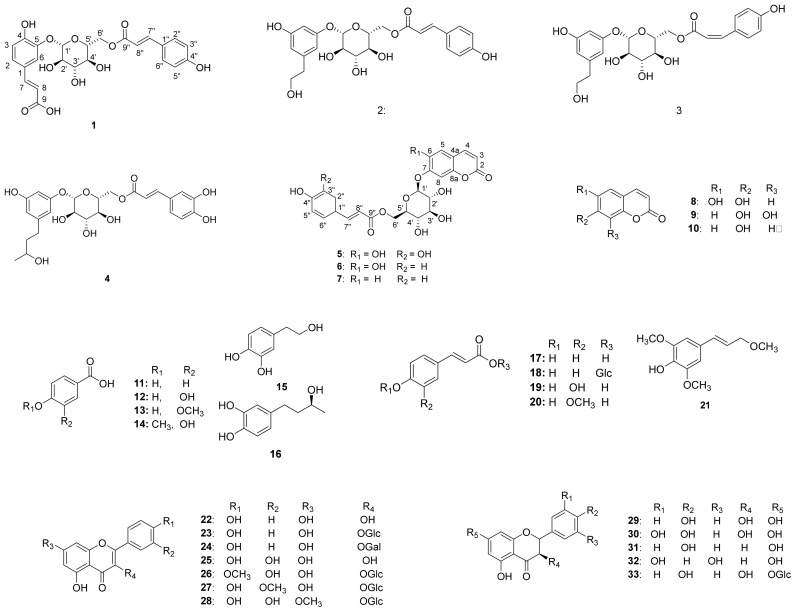

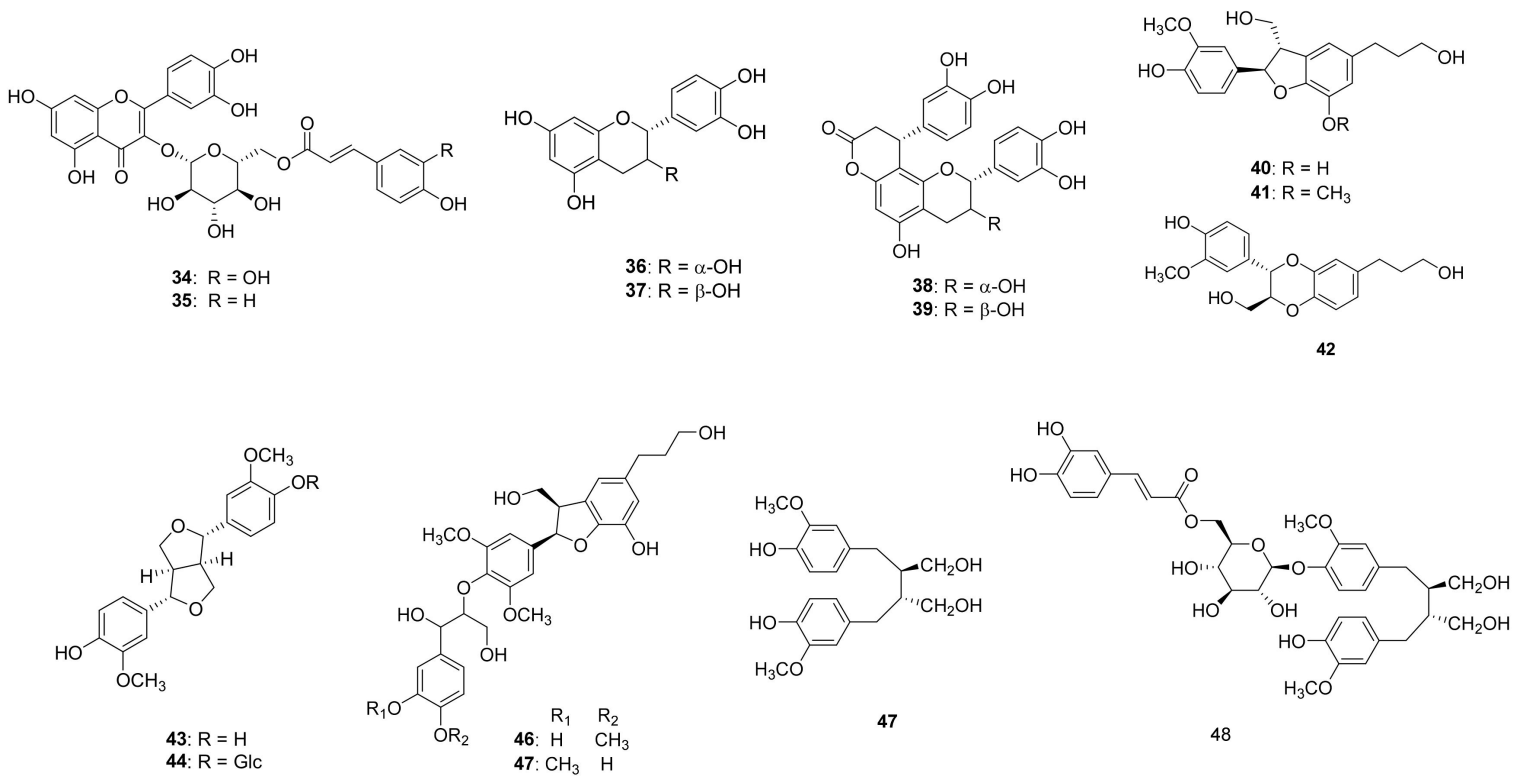
Chemical structures of compounds **1**–**48** from the leaves of *A. arguta*.

Chromatographic separation of the EtOAc fraction of *A. arguta* resulted in the isolation of five new compounds (**1**–**5**) together with forty-three known compounds (**6**–**48**) (Figure 1). 

#### 3.1.1. Structural Determination of New Compounds

**Figure 2:**
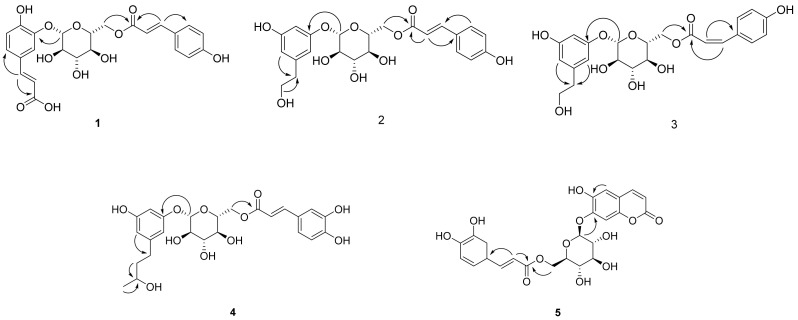
Key HMBC correlations (→) of new compounds **1**–**5**.

Compound **1** (Table 1 and Table 2) was isolated as a brown syrup and the molecular formula was deduced as C_24_H_24_O_11_ from the HRESI-TOF-MS (*m/z* 511.1210 [M + Na]^+^, calcd. for C_24_H_24_NaO_11_, 511.1216) and ^13^C-NMR data. The IR spectrum showed typical absorption bands of hydroxy and carbonyl groups at 3411 and 1664 cm^−1^, respectively. The ^1^H-NMR spectrum of compound **1** showed typical signals for a glucosyl anomeric proton in the β-configuration at *δ*_H_ 4.92 (1H, d, *J* = 7.6 Hz, H-1′). The presence of a glucosyl moiety was also confirmed by the glucosyl carbon signals at 101.9 (C-1′), 73.3 (C-2′), 76.0 (C-3′), 70.6 (C-4′), 74.3 (C-5′), 63.5 (C-6′)]. In the aromatic regions of the ^1^H- and ^13^C-NMR, signals for a 1,3,4-trisubstituted aromatic ring at [*δ*_H_ 7.41 (1H, d, *J* = 2.0 Hz, H-2), 7.17 (1H, d, *J* = 8.4, 2.0 Hz, H-6), 6.88 (1H, d, *J =* 8.4 Hz, H-5); *δ*_C_ 126.6 (C-1), 116.0 (C-2), 145.4 (C-3), 149.4 (C-4), 116.1 (C-5), 124.4 (C-6)] and a 1,4-disubstituted aromatic ring at [*δ*_H_ 7.42 (2H, d, *J* = 8.8 Hz, H-3”, 5”), 6.80 (2H, d, *J* = 8.8 Hz, H-2”, 6”); *δ*_C_ 125.7 (C-1”), 115.4 (C-2”, 6”), 129.9 (C-3”, 5”), 159.9 (C-4”)] were observed. The ^1^H-NMR spectrum also revealed two pairs of olefinic groups in the *trans* configuration at [*δ*_H_ 7.58 (1H, d, *J* = 15.6 Hz, H-7), 6.33 (1H, d, *J =* 15.6 Hz, H-8); *δ*c 144.7 (C-7), 116.1 (C-8)] and [*δ*_H_ 7.61 (1H, d, *J* = 16.0 Hz, H-7”), 6.34 (1H, d, *J =* 16.0 Hz, H-8”); *δ*c 145.7 (C-7”), 113.2 (C-8”)], respectively. Additionally, signals for two carbonyl carbons [*δ*_C_ 169.6 and 167.8] were observed in the ^13^C-NMR spectrum. These signals were assigned to the *trans*-caffeoyl group and the *trans*-coumaroyl group based on the HMBC correlations between H-7/C-1, H-7/C-9 and H-2”/C-7”, H-7”/C-9”, respectively (Figure 2). Therefore, compound **1** was suggested to consist of a glucose, a *trans*-caffeoyl group and a *trans*-coumaroyl group. The connections between these moieties were determined by HMBC correlation. The HMBC correlations from H-1′ of a glucose to C-5 of a *trans*-caffeoyl group, and from H-6′ of a glucose to C-9″ of a *trans*-coumaroyl group suggested the linkages of a *trans*-caffeoyl group to a glucose and a glucose to a *trans*-coumaroyl group. Combined with the above-mentioned data, compound **1** was elucidated as shown and named argutoside A.

Compound **2** (Table 1 and Table 2) was purified as a brown syrup. The molecular formula was deduced as C_23_H_26_NaO_10_ from the HRESI-TOF-MS (*m/z* 485.1418 [M + Na]^+^, calcd. for C_23_H_26_NaO_10_, 485.1424)), which was verified by its ^13^C-NMR data. Similar to compound **1**, the presence of a glucose was easily deduced from glucosyl anomeric signals [*δ*_H_ 4.78 (1H, d, *J* = 7.6 Hz, H-1′); *δ*_C_ 102.9]. The presence of a *trans*-coumaroyl group was also suggested by the signals at [*δ*_H_ 6.83 (2H, d, *J* = 8.8 Hz, H-2”, 6”), 7.50 (2H, d, *J* = 8.8 Hz, H-3”, 5”), 7.68 (1H, d, *J* = 16.0 Hz, H-7”), 6.41 (1H, d, *J =* 16.0 Hz, H-8”); *δ*_C_ 125.7 (C-1”), 115.4 (C-2”, 6”), 129.9 (C-3”, 5”), 160.0 (C-4”), 145.6 (C-7”), 113.4 (C-8”), 167.6 (C-9”)] together with the HMBC correlations. Besides the aforementioned signals for a glucose and *trans*-coumaroyl group, signals for a 1,3,5-trisubstituted aromatic ring [*δ*_H_ 6.78 (2H, s, H-2, 4), 7.04 (1H, s, H-6); *δ*_C_ 130.7 (C-1), 123.9 (C-2), 145.1 (C-3), 115.5 (C-4), 145.3 (C-5), 118.1 (C-6)] two methylene [*δ*_H_ 2.67 (2H, t, *J* = 7.2 Hz, H-7), 3.67 (2H, t, *J* = 7.2 Hz, H-8); *δ*_C_ 38.2 (C-7), 62.9 (C-8)], and an oxygenated methine [*δ*_H_ 3.62 (1H, m, H-9); *δ*_C_ 66.3] were observed in ^1^H- and ^13^C-NMR together with HSQC spectrum. These additional signals were assigned to a 3, 5-dihydroxyphenylethanol group based on the correlations between H-2/C-7 and H-8/C-7 in the HMBC spectrum. The positions of a 3, 5-dihydroxyphenylethanol group and a *trans*-coumaroyl group were determined to be C-1′ and C-6′, respectively, from the HMBC correlations of H-1′/C-3 and H-6′/C-9”. Consequently, the structure of compound **2** was defined as shown and named argutoside B. The ^1^H- and ^13^C-NMR spectra of **3** (Table 1 and Table 2) were similar to those of compound **2**, with the difference being the replacement of the *trans*-olefinic protons with a large coupling constant [*δ*_H_ 7.68 (1H, d, *J* = 16.0 Hz, H-7”), 6.41 (1H, d, *J =* 16.0 Hz, H-8”)] by *cis*-olefinic protons with a smaller coupling constant [*δ*_H_ 6.93 (1H, d, *J* = 12.8 Hz, H-7′), 5.86 (1H, d, *J =* 12.8 Hz, H-8′)]. Therefore, the structure of compound **3** was defined as shown and named argutoside C. 

Compound **4** (Table 1 and Table 2) was purified as brown syrup. The molecular formula was deduced as C_25_H_30_NaO_11_ from the HRESI-TOF-MS (*m/z* 529.1680 [M + Na]^+^, calcd. for C_25_H_30_NaO_11_, 529.1686), which was verified by its ^13^C-NMR data. Similar to compounds 1–3, the presence of a glucose was easily deduced from glucosyl anomeric signals [*δ*_H_ 4.77 (1H, d, *J* = 7.2 Hz, H-1′); *δ*_C_ 102.9]. The presence of a *trans*-caffeoyl group was also suggested from the signals at [*δ*_H_ 7.08 (1H, d, *J* = 1.6 Hz, H-2”), 6.80 (1H, d, *J* = 8.4, 1.6 Hz, H-5”), 6.97 (1H, d, *J =* 8.4 Hz, H-6”), 7.60 (1H, d, *J* = 15.6 Hz, H-7”), 6.33 (1H, d, *J =* 15.6 Hz, H-8”); *δ*_C_ 126.2 (C-1”), 113.7 (C-2”), 145.5 (C-3”), 148.3 (C-4”), 115.1 (C-5”), 121.8 (C-6”), 146.0 (C-7”), 113.3 (C-8”), 167.6 (C-9”)] together with the HMBC correlations. Besides the aforementioned signals, signals for a 1,3,5-trisubstituted aromatic ring [*δ*_H_ 6.76 (2H, s, H-2, 4), 7.00 (1H, s, H-6); *δ*_C_ 133.9 (C-1), 123.2 (C-2), 144.8 (C-3), 115.5 (C-4), 145.1 (C-5), 117.5 (C-6)], two methylene [*δ*_H_ 2.53 (2H, m, H-7), 1.63 (2H, m, H-8); *δ*_C_ 31.1 (C-7), 40.6 (C-8)], an oxygenated methine [*δ*_H_ 3.62 (1H, m, H-9); *δ*_C_ 66.3] and a methyl group [*δ*_H_ 1.11 (3H, d, *J* = 6.0 Hz, H-10); *δ*_C_ 22.1] were observed in ^1^H- and ^13^C-NMR together with the HSQC spectrum. The HMBC correlations of H-2/C7, H-8/C-9 and H-10/C-9 attributed the connection of a 1-(3,5-dihydroxyphenyl)-butan-3-ol group to the glucose and *trans*-caffeoyl groups. The HMBC correlations of H-1′/C-3 and of H-6′/C-9″ confirmed the linkage between a *trans*-caffeoyl group, a glucose and a 1-(3,5-dihydroxyphenyl)-butan-3-ol group, as shown in Figure 2. Conclusively, compound **4** was defined as shown and named argutoside D.

Compound **5** (Table 3) was purified as a brown syrup with the molecular formula of C_24_H_22_O_12_ deduced by HRESI-TOF-MS analysis (*m/z* 525.1003, calcd. for C_24_H_22_NaO_12_, 525.1009) and ^13^C-NMR data. The ^1^H- and ^13^C-NMR spectra of compound **5** showed the signals for a glucose and a *trans*-coumaroyl group, similar to those of compounds **1**–**4**. However, signals for two *cis*-olefinic protons at *δ*_H_ 7.68 (1H, d, *J* = 9.2 Hz, H-4) and 5.85 (1H, d, *J* = 9.2 Hz, H-3) suggested that compound **5** was a coumarin derivative, which was supported by the characteristic UV absorption maxima at 211 and 327 nm. Additionally, two aromatic protons at *δ*_H_ 7.24 (1H, s, H-5) and *δ*_H_ 6.74 (1H, s, H-8) together with ^13^C-NMR signals of *δ*_C_ 166.8 (C-2), 111.7 (C-3), 144.7 (C-4), 110.6 (C-4a), 114.7 (C-5), 151.1 (C-6), 143.2 (C-7), 103.7 (C-8), 146.1 (C-8a)] suggested the existence of one 6,7-disubstituted coumarin skeleton. Further analysis using the HMBC correlation together with a comparison to the previous data identified the coumarin moiety as 6,7-dihydroxycoumarin, esculetin [22]. Therefore, compound **1** was suggested to consist of a glucose, a *trans*-caffeoyl group and a 6,7-dihydroxycoumarin moiety. The HMBC correlations from H-1′ of a glucose to C-7 of a 6,7-dihydroxycoumarin group suggested the linkage between 6,7-dihydroxycoumarin and a glucose, and the HMBC correlations from H-6′ of a glucose to C-9″ of *trans*-caffeoyl group suggested the linkage between a glucose and a *trans*-caffeoyl group. Based on these data, the structure of compound **5** was defined as esculetin 7-*O*-(6′-*O*-*trans*-caffeoyl)-*β*-glucopyranoside and named argutoside E.

#### 3.1.2. Identification of Known Compounds

The known compounds were identified as esculetin 7-*O*-(6′-*O*-*trans*-coumaroyl)-*β*-glucopyranoside (**6**) [23], umbelliferone 7-*O*-(6′-*O*-*trans*-coumaroyl)-*β*-glucopyranoside (**7**) [23], esculetin (**8**) [22], 7,8-Dihydroxycoumarin (**9**) [24], umbelliferone (**10**) [25], 4-hydroxybenzoic acid (**11**) [26], protocatechuic acid (**12**) [26], vanillic acid (**13**) [27], isovanillic acid (**14**) [28], hydroxytyrosol (**15**) [28], (-)-rhodolatouchol (**16**) [29], *p*-*E*-coumaric acid (**17**) [30], *p*-*E*-coumaric acid-9-*O*-glucopyranoside (**18**) [23], *E*-caffeic acid (**19**) [28], *E*-ferulic acid (**20**) [31], 3,5-dimethoxy-4-hydroxycinnamic alcohol (**21**) [32], kaempferol (**22**) [33], kaempferol 3-*O-β*-glucopyranoside (**23**) [34], kaempferol 3-*O-β*-galactopyranoside (**24**) [35], quercetin (**25**) [36], tamarixin (**26**) [37], isorhamnetin 3-*O-β*-glucopyranoside (**27**) [30], rhamnetin 3-*O-β*-glucopyranoside (**28**) [35], dihydrokaempferol (**29**) [35], dihydroquercetin (**30**) [38], naringenin (**31**) [35], 5,7,3′,5′-tetrahydroxyflavanone (**32**) [38], sinensin (**33**) [39], quercetin 3-*O*-(6”-*O*-*E*-caffeoyl)-*β-*glucopyranoside (**34**) [39], quercetin 3-*O*-(6”-*O*-*E*-coumaroyl)-*β*-glucopyranoside (**35**) [40], epicatechin (**36**) [30], catechin (**37**) [35], cinchonain Ia (**38**) [41], catechin-[8,7-e]-4b-(3,4-dihydroxy-phenyl)-dihydro-2(3*H*)-pyranone (**39**) [40], 7*S*,8*R*-cedrusin (**40**) [42], dehydroconiferyl alcohol (**41**) [40], (7*S*,8*S*)-3-methoxy-3′,7-epoxy-8,4′-oxyneoligna-4,9,9′-triol (**42**) [43], pinoresinol (**43**) [44], pinoresinol 4-*O-β-*glucopyranoside (**44**) [42], alutaceuol (**45**) [29], alutaceuol isomer (**46**) [29], (-)-(2*R*,3*R*)-secoisolariciresinol (**47**) [45], glehlinoside F (**48**) [46] via analysis of their physical data and comparison with literature values.

### 3.2. Antioxidant and α-Glucosidase Inhibitory Activity

#### 3.2.1. Antioxidant and α-Glucosidase Inhibitory Activity of Compounds

**Figure 3:**
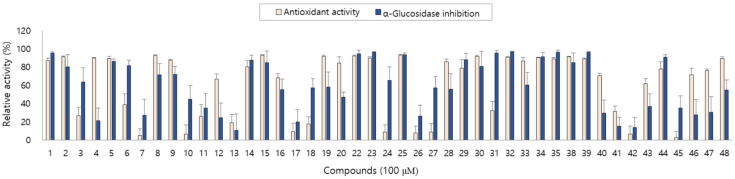
Antioxidant and α-glucosidase inhibitory activity of compounds **1**–**48** from *A. arguta* leaves.

The antioxidant and anti-diabetic activity of the isolated compounds were evaluated by measuring the DPPH radical scavenging and α-glucosidase inhibitory activity. The isolated compounds showed good antioxidant and α-glucosidase inhibitory activity with differences in activity depending on the structures. In particular, new compounds **1**, **2**, **4** and **5** showed antioxidant activity and compounds **1**, **3** and **5** showed α-glucosidase inhibitory activity in our assay system (Figure 3).

As described above, the leaf of *A. arguta* is rich in phenolic compounds, and a total of 48 compounds were purified in this study. All 48 of the compounds that were isolated in this study are aromatic compounds and can be subdivided according to the compound skeleton as follows: phenylpropanoid derivatives (**1**–**4**, **16**–**21**), coumarins (**5**–**10**), simple phenolics (**11**–**15**), flavonoids (**22**–**39**) and lignans (**40**–**48**). The biological activity of the isolated compounds differs depending on their structure, and flavonoid showed excellent efficacy while lignan showed comparatively weak efficacy in our assay system. Interestingly, the leaf of *A. arguta* contained phenolic-conjugates that were bound to various skeletons such as phenylpropanoid-conjugates, coumarin-conjugates, flavonoid-conjugates and lignan-conjugates. These conjugates showed antioxidant and α-glucosidase inhibitory activity and contributed to the beneficial effect of *A. arguta* leaves.

#### 3.2.2. Molecular Docking Analysis

**Figure 4:**
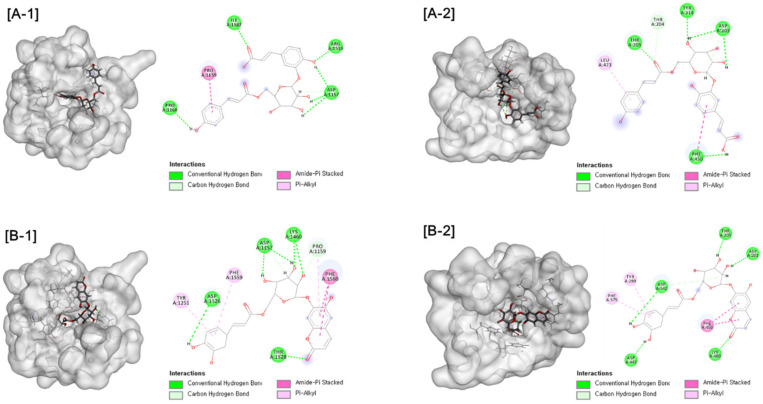
[A] Docking picture of compound **1** to CtMGAM (A-1) and NtMGAM (A-2) and [B] compound **5** to CtMGAM (B-1) and NtMGAM (B-2). The interactions of conventional hydrogen bond (green color), carbon hydrogen bond (light green color), amide-Pi stacked (pink color) and Pi-alkyl (light pine) were shown.

Further molecular docking analysis was conducted for two types of human maltase-glucoamylase (NtMGAM and CtMGAM) in order to propose the mechanisms of the α-glucosidase inhibitory activity of active compounds. Consistent with experimental results, interactions with the α-glucosidase were suggested for active compounds. Hydrogen bonds were formed between compound **1** and NtMGAM and CtMGAM, respectively. Compound **5** exhibited the interaction by forming hydrogen bonds and Pi-alkyl interactions, as shown in Figure 4. These results indicate that compounds **1** and **5** could be inserted into the active site of the enzyme by different types of interactions and could inhibit α-glucosidase activity.

## 4. Conclusions

An investigation into the leaves of *A. arguta* led to the isolation of 48 aromatic compounds, including 5 new compounds. The structures of the isolated compounds were determined to be aromatic, including phenylpropanoid derivatives, phenolics, coumarins, flavonoids and lignans. Five new compounds were defined as argutosides A–D (**1**–**4**), which consist of phenylpropanoid glycosides conjugated with a phenolic compound, and argutosides E (**5**), which is a coumarin glycoside conjugated with a phenylpropanoid. The isolated compounds showed good antioxidant and α-glucosidase inhibitory activity with differences in activity depending on the structures. The analysis of the interactions between hydroxyl and carbonyl groups of active compounds **1** and **5** and α-glucosidase by molecular docking analysis supported the α-glucosidase inhibitory activity. In conclusion, the aromatic constituents of *A. arguta* leaves with α-glucosidase inhibitory activity might be beneficial to glucose-related diseases. 

## Figures and Tables

**Table 1:** ^1^H-NMR spectroscopic data for compounds **1**–**4** (CD_3_OD).

Title 1	1	2	3	4
2	7.41 (d, 2.0)	6.78 (s)	6.78 (s)	6.76 (s)
4	-	6.78 (s)	6.78 (s)	6.76 (s)
5	6.88 (d, 8.4)	-	-	-
6	7.17 (dd, 8.4, 2.0)	7.04 (s)	7.04 (s)	7.00 (s)
7	7.58 (d, 15.6)	2.67 (2H, t, 7.2)	2.71 (2H, t, 7.2)	2.53 (2H, m)
8	6.33 (d, 15.6)	3.67 (2H, t, 7.2)	3.70 (2H, t, 7.2)	1.63 (2H, m)
9	-	-	-	3.62 (m)
10	-	-	-	1.11 (3H, d, 6.0)
1′	4.92 (d, 7.6)	4.78 (d, 7.6)	4.76 (d, 7.6)	4.77 (d. 7.2)
2′	3.41–3.57 (m)	3.40–3.54 (m)	3.40–3.52 (m)	3.41–3.54 (m)
3′	3.41–3.57 (m)	3.40–3.54 (m)	3.40–3.52 (m)	3.41–3.54 (m)
4′	3.41–3.57 (m)	3.40–3.54 (m)	3.40–3.52 (m)	3.41–3.54 (m)
5′	3.82 (m)	3.73 (m)	3.67 (m)	3.74 (m)
6′	4.59 (dd, 12.0, 2.0)	4.60 (dd, 12.0, 2.0)	4.55 (dd, 12.0, 2.0)	4.59 (dd, 12.0, 2.4)
	4.38 (dd, 12.0, 6.6)	4.37 (dd, 12.0, 6.6)	4.33 (dd, 12.0, 6.6)	4.37 (dd, 12.0, 6.8)
2′′	6.80 (d, 8.8)	6.83 (d, 8.8)	6.73 (d, 8.8)	7.08 (d, 1.6)
3′′	7.42 (d, 8.8)	7.50 (d, 8.8)	7.66 (d, 8.8)	-
5′′	7.42 (d, 8.8)	7.50 (d, 8.8)	7.66 (d, 8.8)	6.80 (d, 8.4)
6′′	6.80 (d, 8.8)	6.83 (d, 8.8)	6.73 (d, 8.8)	6.97 (dd, 8.4, 1.6)
7′′	7.61 (d, 16.0)	7.68 (d, 16.0)	6.93 (d, 12.8)	7.60 (d, 15.6)
8′′	6.34 (d, 16.0)	6.41 (d, 16.0)	5.83 (d, 12.8)	6.33 (d, 15.6)

**Table 2:** ^13^C-NMR spectroscopic data for compounds **1**–**4** (CD_3_OD).

Carbon NO.	1	2	3	4
1	126.6	130.7	130.6	133.9
2	116.0	123.9	123.9	123.2
3	145.4	145.1	145.1	144.8
4	149.4	115.5	115.5	115.5
5	116.1	145.3	145.3	145.1
6	124.4	118.1	118.1	117.5
7	144.7	38.2	38.2	31.1
8	115.5	62.9	63.0	40.6
9	169.6	-	-	66.3
10	-	-	-	22.1
1′	101.9	102.9	102.9	102.9
2′	73.3	73.4	73.4	73.4
3′	76.0	76.0	76.0	76.0
4′	70.6	70.4	70.3	70.4
5′	74.3	74.4	74.3	74.4
6′	63.5	63.3	63.0	63.3
1”	125.7	125.7	126.1	126.2
2”	115.4	115.4	114.4	113.7
3”	129.9	129.9	132.4	145.5
4”	159.9	160.0	158.7	148.3
5”	129.9	129.9	132.4	115.1
6”	115.4	115.4	114.4	121.8
7”	145.7	145.6	144.3	146.0
8”	113.2	113.4	114.7	113.3
9”	167.8	167.6	166.6	167.6

**Table 3:** ^1^H- and ^13^C-NMR spectroscopic data for compound **5** (DMSO-*d_6_*).

Carbon NO.	^1^H	^13^C
2	-	166.8
3	5.85 (d, 9.2)	111.7
4	7.68 (d, 9.2)	144.7
4a	-	110.6
5	7.24 (s)	114.7
6	-	151.1
7	-	143.2
8	6.74 (s)	103.7
8a	-	146.1
1′	4.81 (d, 7.2)	102.3
2′	3.34–3.50 (m)	73.6
3′	3.34–3.50 (m)	76.3
4′	3.34–3.50 (m)	70.5
5′	3.68 (m)	74.5
6′	4.45 (dd, 12.0, 1.6), 4.24 (dd, 12.0, 7.2)	63.8
1”	-	125.9
2”	7.06 (d, 1.6)	115.4
3”	-	146.1
4”	-	149.0
5”	6.77 (d, 8.0)	116.2
6”	6.99 (dd, 8.0, 1.6)	121.9
7”	7.49 (d, 16.0)	145.8
8”	6.32 (d, 16.0)	114.3
9”	-	166.8

## Data Availability

The data is contained within the article or Supplementary Material.

## Supplementary Materials

The following are available online at https://www.mdpi.com/article/10.3390/antiox10121896/s1, Figures S1–S25: ^1^H, ^13^C, HSQC, HMBC and HRESI-MS spectrums of new compounds **1**–**5**.
