# Supplementary material for: Aromatic Constituents from the Leaves of Actinidia arguta with Antioxidant and α-Glucosidase Inhibitory Activity
Source: Antioxidants (Basel). 2021 Nov 26;10(12):1896. doi: 10.3390/antiox10121896 (PMC8750355; doi:10.3390/antiox10121896)
Supplement: Supplementary file 1 [file antioxidants-10-01896-s001.zip › antioxidants-1468836-supple final.pdf]

Supplementary Materials

Aromatic Constituents of from the Leaves of *Actinidia arguta* with Antioxidant and α-Glucosidase Inhibitory Activity

Jong Hoon Ahn [1], Se Hwan Ryu [1], Solip Lee [1], Sang Won Yeon [1], Ayman Turk [1], Yoo Kyong Han [2], Ki Yong Lee [2], Bang Yeon Hwang [1] and Mi Kyeong Lee [1,*]

[1] College of Pharmacy, Chungbuk National University, Cheongju 28160, Korea; zzonggoo07@naver.com (J.H.A.); alfm0188@naver.com (S.H.R.);dudaos000@hanmail.net (S.L.); sangwon1311@naver.com (S.W.Y.); ayman.turk@hotmail.com (A.T.); byhwang@chungbuk.ac.kr (B.Y.H.)

[2] College of Pharmacy, Korea University, Sejong 47236, Republic of Korea; kkoo_@naver.com (Y.K.H.); kylee11@korea.ac.kr (K.Y.L.)

* Correspondence: mklee@chungbuk.ac.kr; Tel.: +82-43-261-2818

.

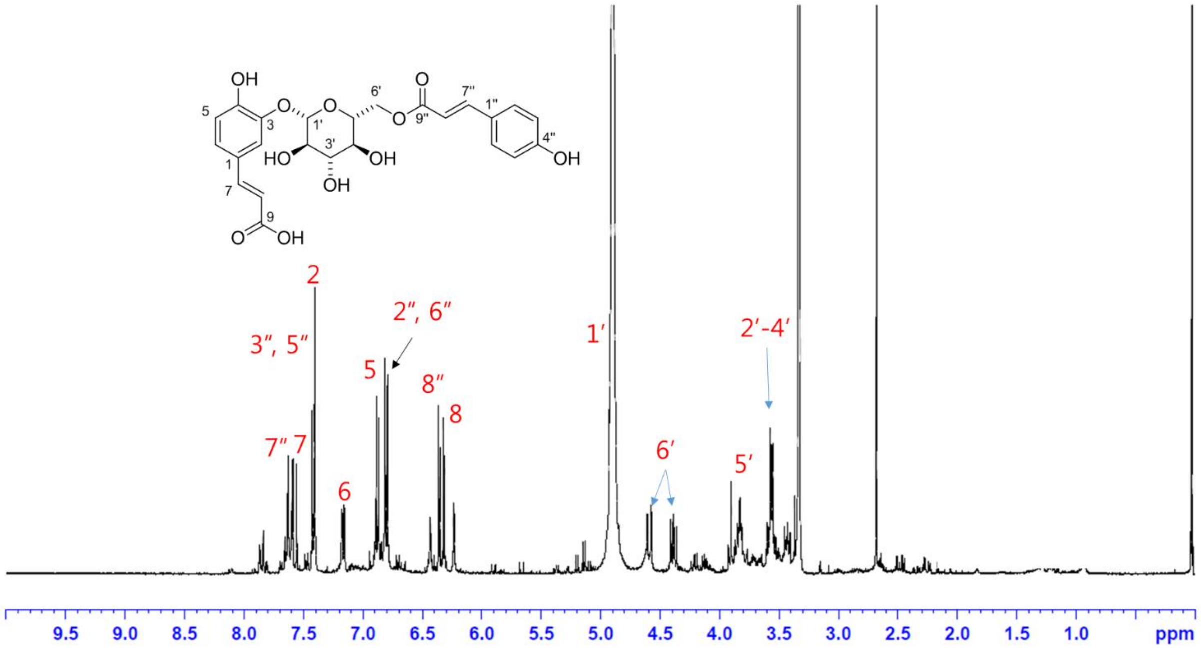

Figure S1. ^{1}H-NMR spectrum of compound **1** (CD_3OD, 400 MHz)

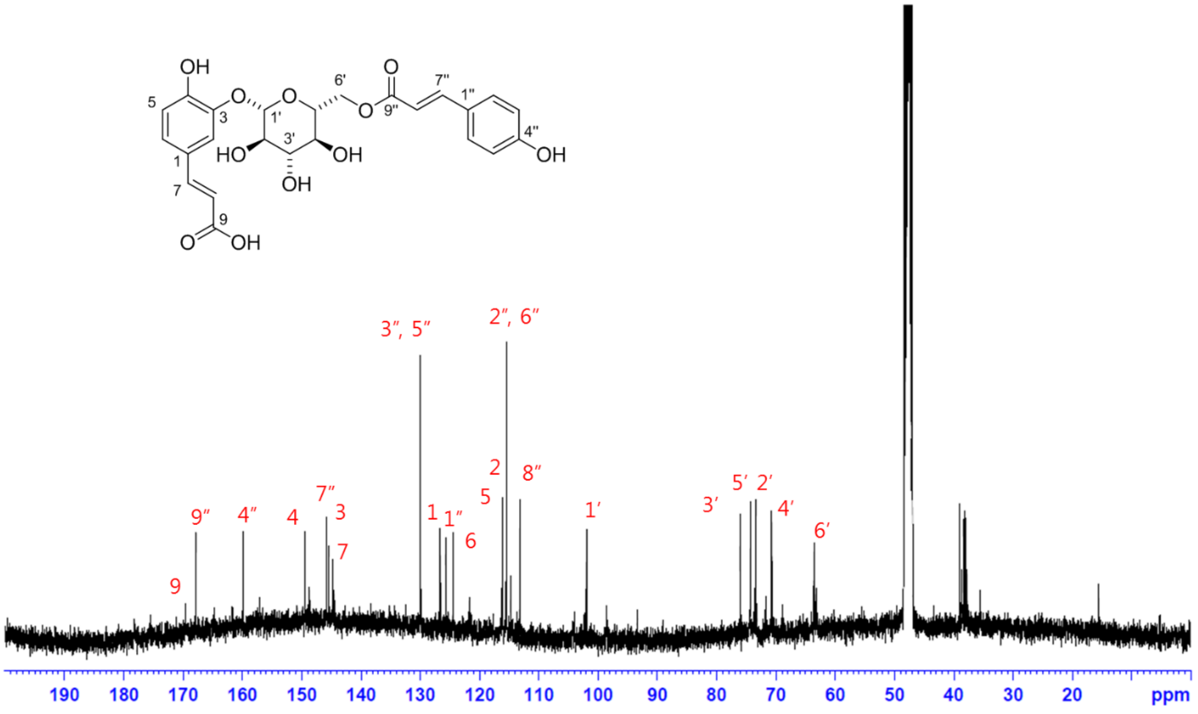

Figure S2. ^{13}C-NMR spectrum of compound **1** (CD_3OD, 100 MHz)

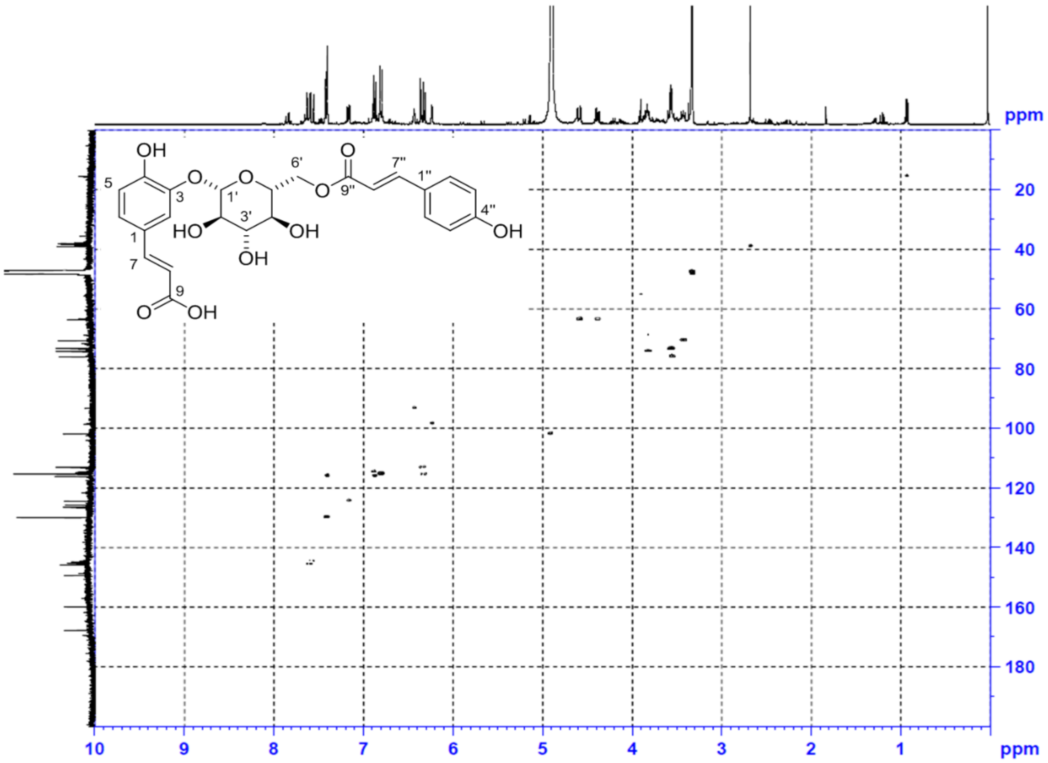

Figure S3. HSQC spectrum of compound **1** (CD_3OD, 100 MHz)

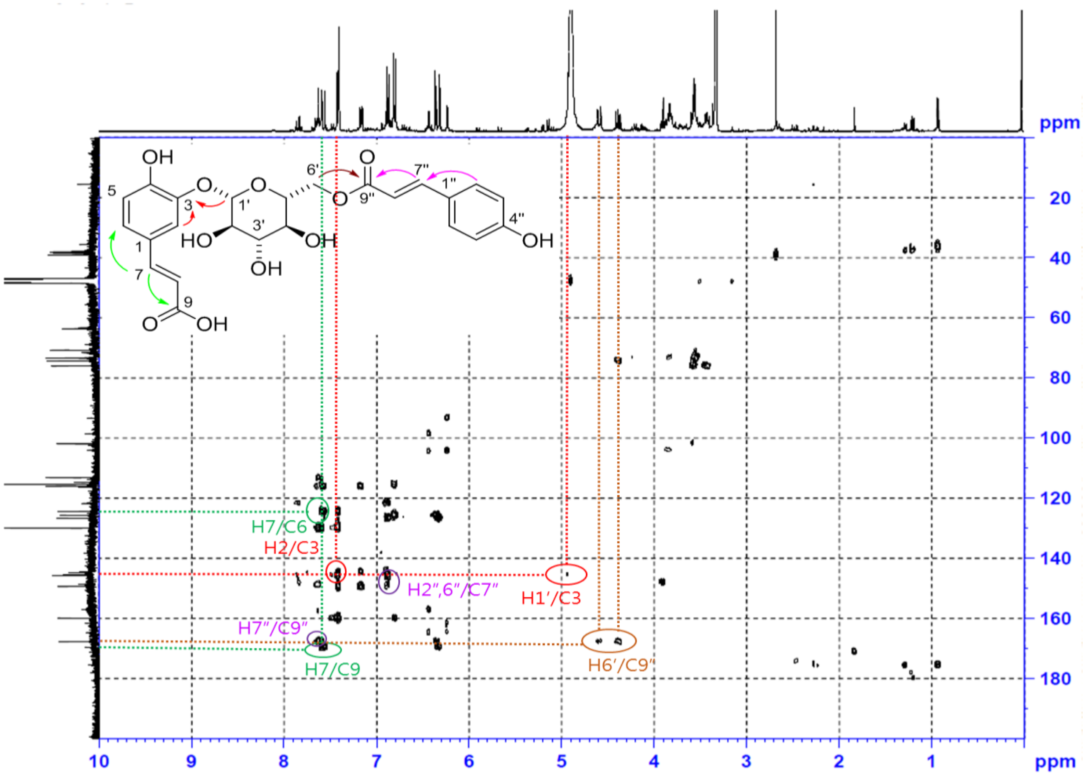

Figure S4. HMBC spectrum of compound **1** (CD_3OD, 100 MHz)

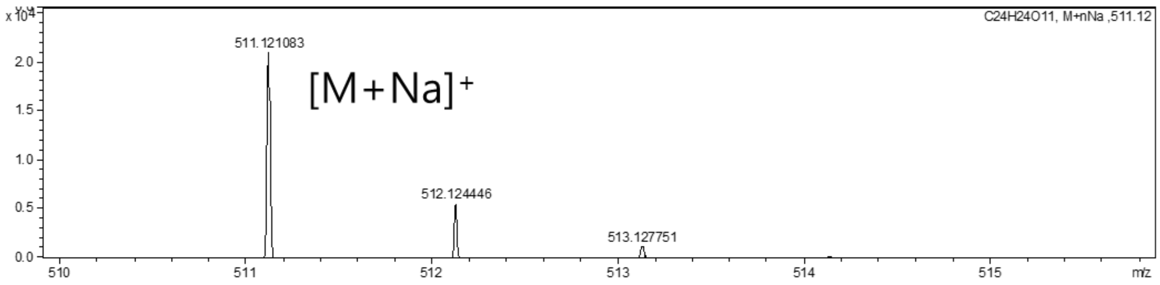

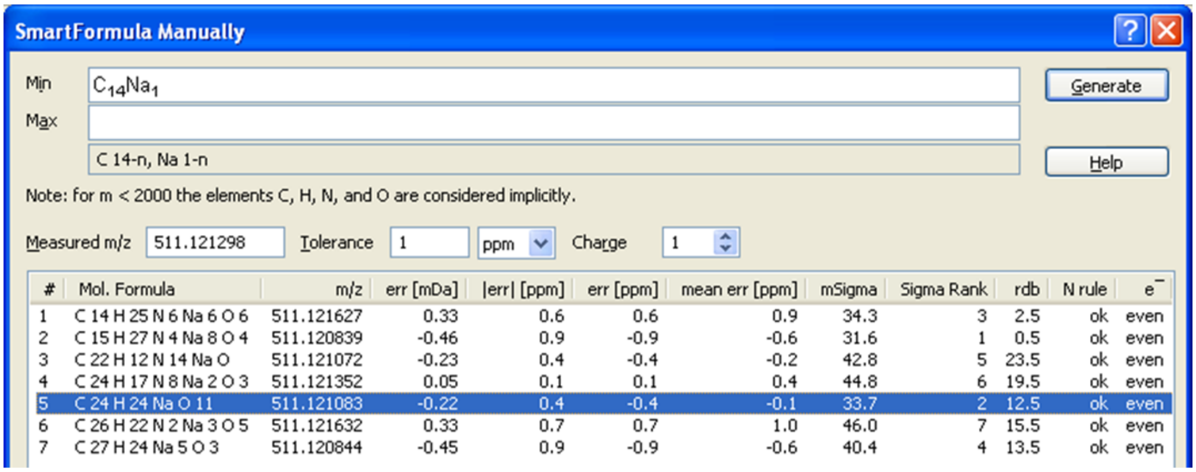
SmartFormula Manually

Min: $C_{14}Na_1$

Max:

C 14-n, Na 1-n

Generate

Help

Note: for m < 2000 the elements C, H, N, and O are considered implicitly.

Measured m/z: 511.121298 Tolerance: 1 ppm Charge: 1

#	Mol. Formula	m/z	err [mDa]	|err| [ppm]	err [ppm]	mean err [ppm]	mSigma	Sigma Rank	rdb	N rule	e⁻
1	C 14 H 25 N 6 Na 6 O 6	511.121627	0.33	0.6	0.6	0.9	34.3	3	2.5	ok	even
2	C 15 H 27 N 4 Na 8 O 4	511.120839	-0.46	0.9	-0.9	-0.6	31.6	1	0.5	ok	even
3	C 22 H 12 N 14 Na O	511.121072	-0.23	0.4	-0.4	-0.2	42.8	5	23.5	ok	even
4	C 24 H 17 N 8 Na 2 O 3	511.121352	0.05	0.1	0.1	0.4	44.8	6	19.5	ok	even
5	C 24 H 24 Na O 11	511.121083	-0.22	0.4	-0.4	-0.1	33.7	2	12.5	ok	even
6	C 26 H 22 N 2 Na 3 O 5	511.121632	0.33	0.7	0.7	1.0	46.0	7	15.5	ok	even
7	C 27 H 24 Na 5 O 3	511.120844	-0.45	0.9	-0.9	-0.6	40.4	4	13.5	ok	even

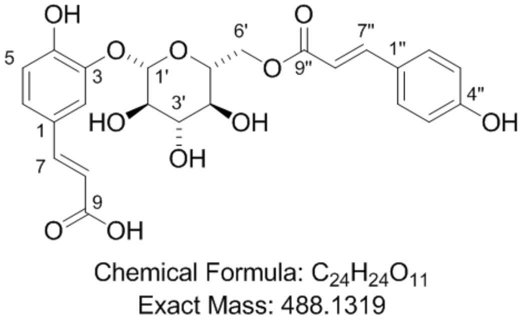

HRESI-TOF-MS
m/z 511.1210
(calcd. for $C_{24}H_{24}NaO_{11}$ 511.1216)

Figure S5. HRESI-TOF-MS spectrum of compound **1**

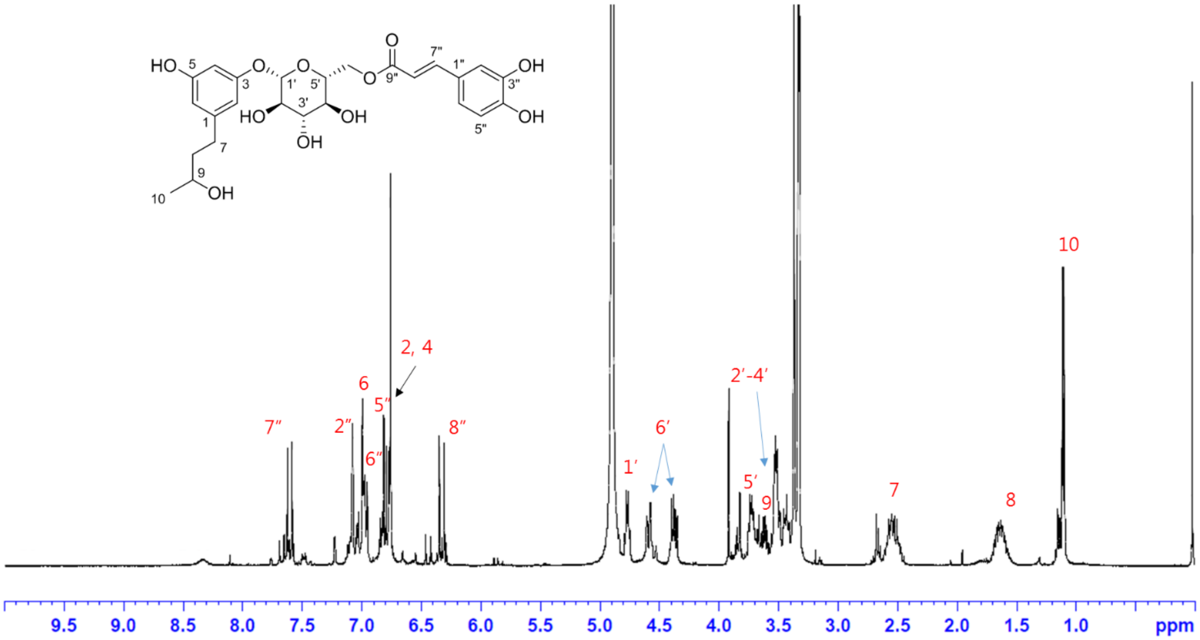

Figure S6. ^{1}H-NMR spectrum of compound **2** (CD_3OD, 400 MHz)

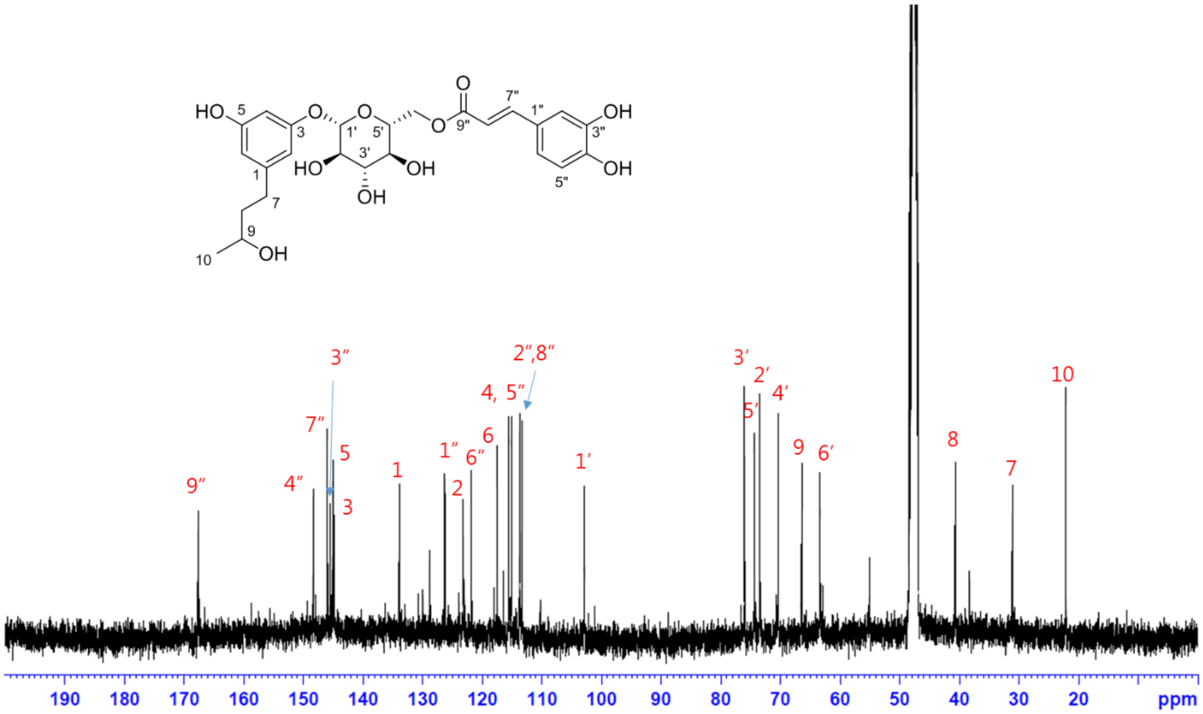

Figure S7. ^{13}C-NMR spectrum of compound **2** (CD_3OD, 100 MHz)

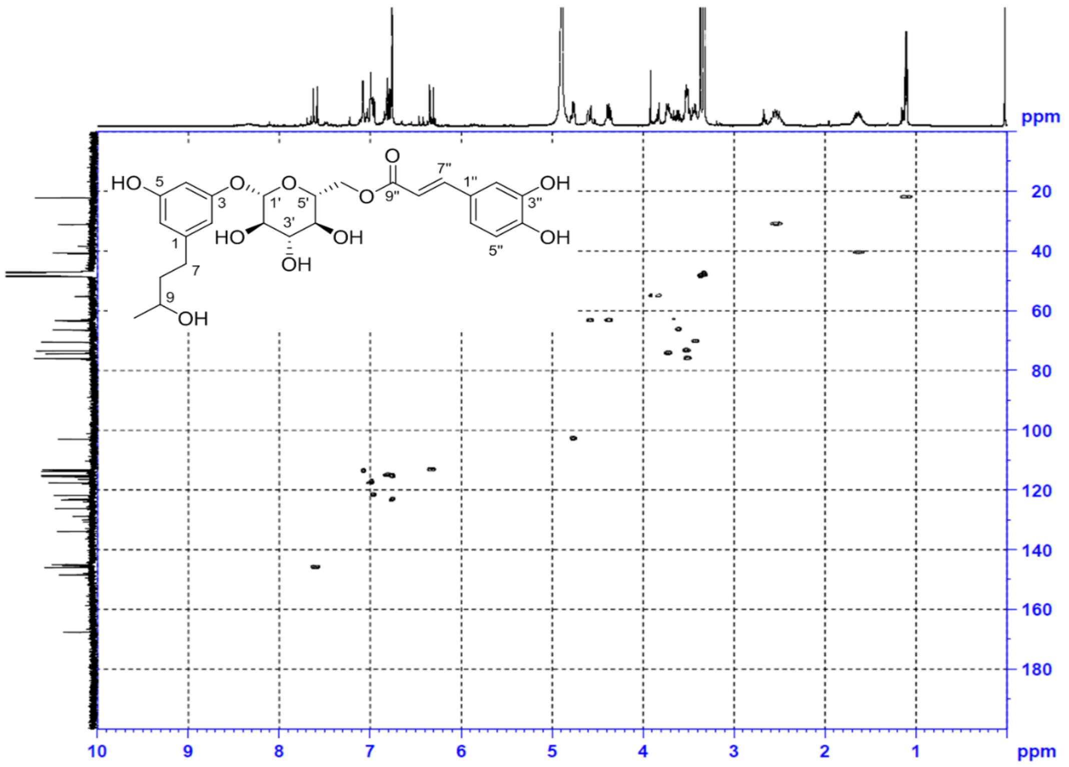

Figure S8. HSQC spectrum of compound **2** (CD_3OD, 100 MHz)

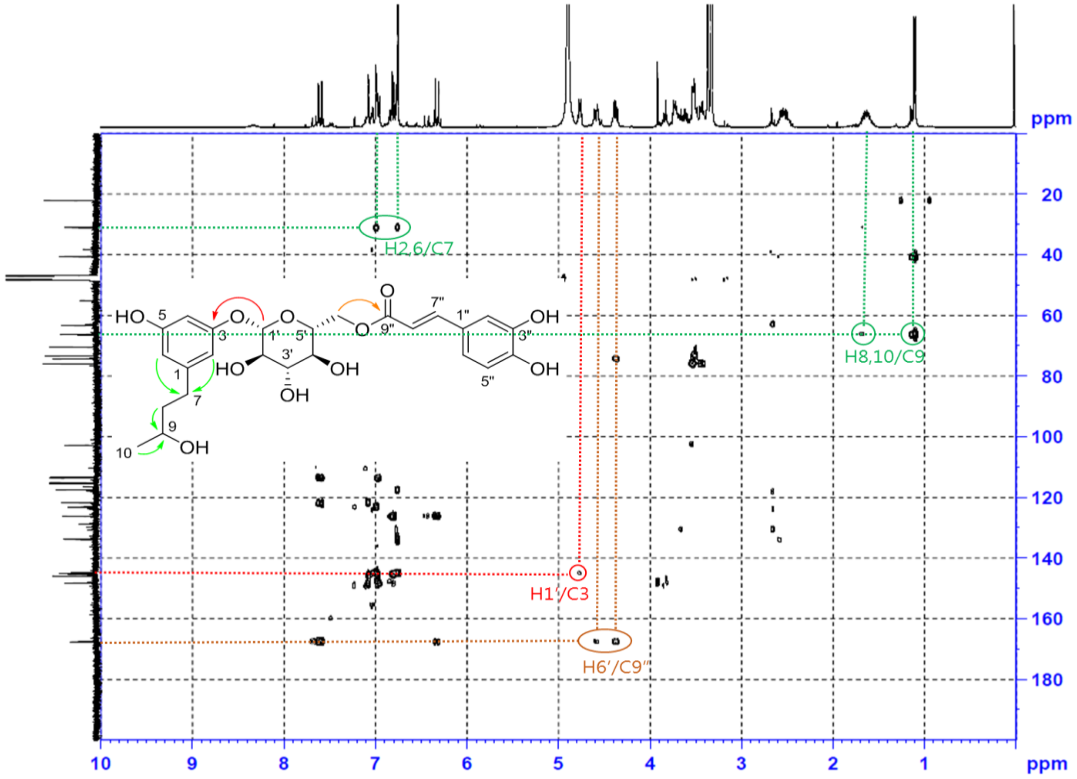

Figure S9. HMBC spectrum of compound **2** (CD_3OD, 100 MHz)

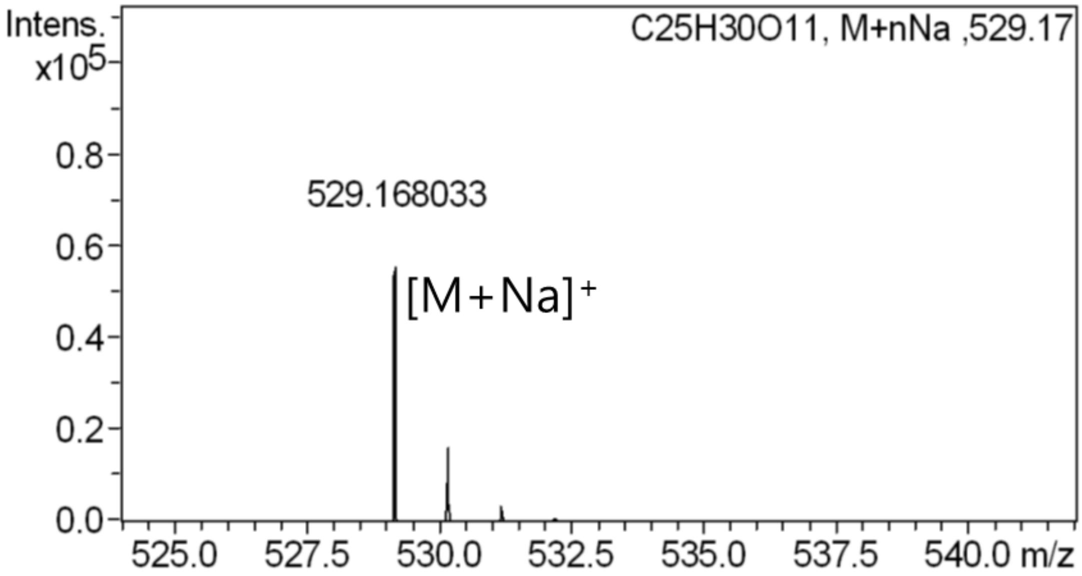

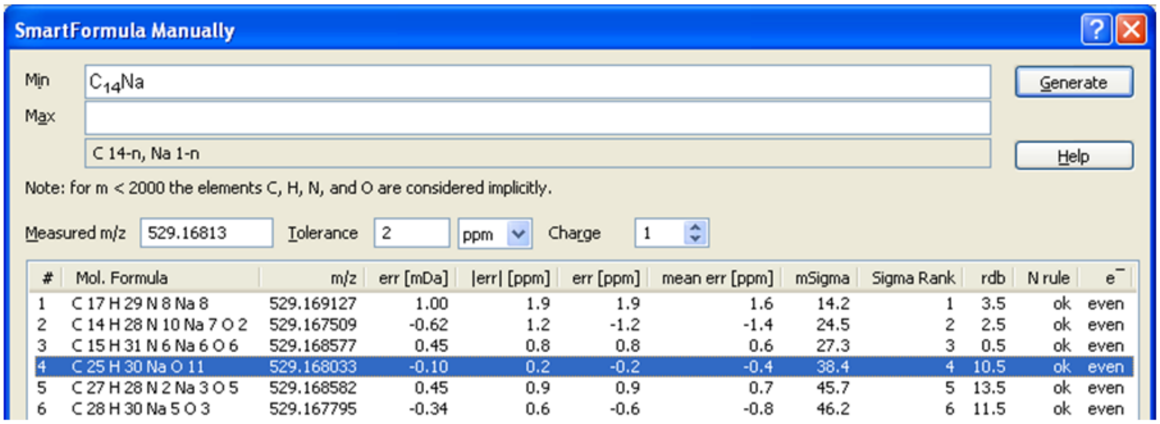

#	Mol. Formula	m/z	err [mDa]	|err| [ppm]	err [ppm]	mean err [ppm]	mSigma	Sigma Rank	rdb	N rule	e⁻
1	C 17 H 29 N 8 Na 8	529.169127	1.00	1.9	1.9	1.6	14.2	1	3.5	ok	even
2	C 14 H 28 N 10 Na 7 O 2	529.167509	-0.62	1.2	-1.2	-1.4	24.5	2	2.5	ok	even
3	C 15 H 31 N 6 Na 6 O 6	529.168577	0.45	0.8	0.8	0.6	27.3	3	0.5	ok	even
4	C 25 H 30 Na O 11	529.168033	-0.10	0.2	-0.2	-0.4	38.4	4	10.5	ok	even
5	C 27 H 28 N 2 Na 3 O 5	529.168582	0.45	0.9	0.9	0.7	45.7	5	13.5	ok	even
6	C 28 H 30 Na 5 O 3	529.167795	-0.34	0.6	-0.6	-0.8	46.2	6	11.5	ok	even

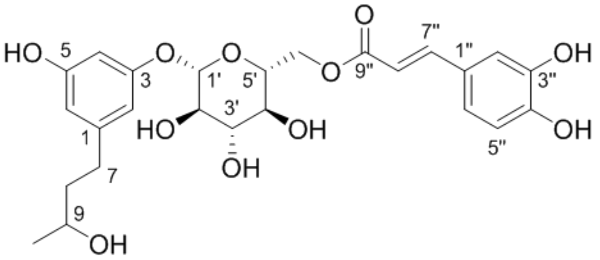

Chemical Formula: $C_{25}H_{30}O_{11}$
Exact Mass: 506.1788

HRESI-TOF-MS
m/z 529.1680
(calcd. for $C_{25}H_{30}NaO_{11}$ 529.1686)

Figure S10. HRESI-TOF-MS spectrum of compound **2**

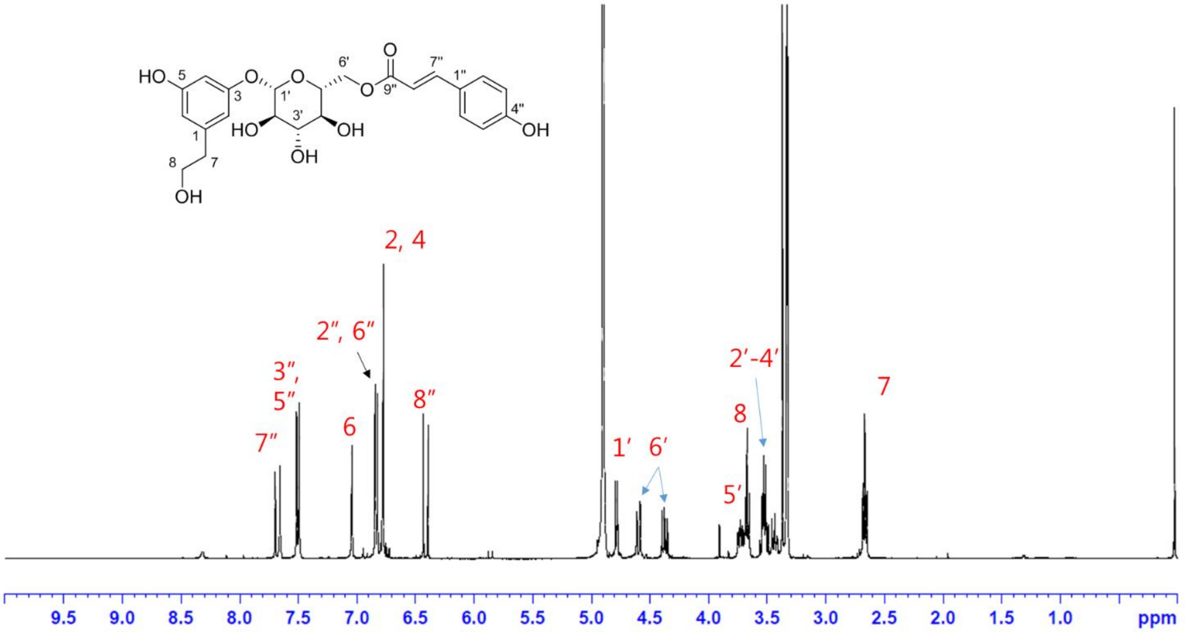

Figure S11. ^{1}H-NMR spectrum of compound **3** (CD_3OD, 400 MHz)

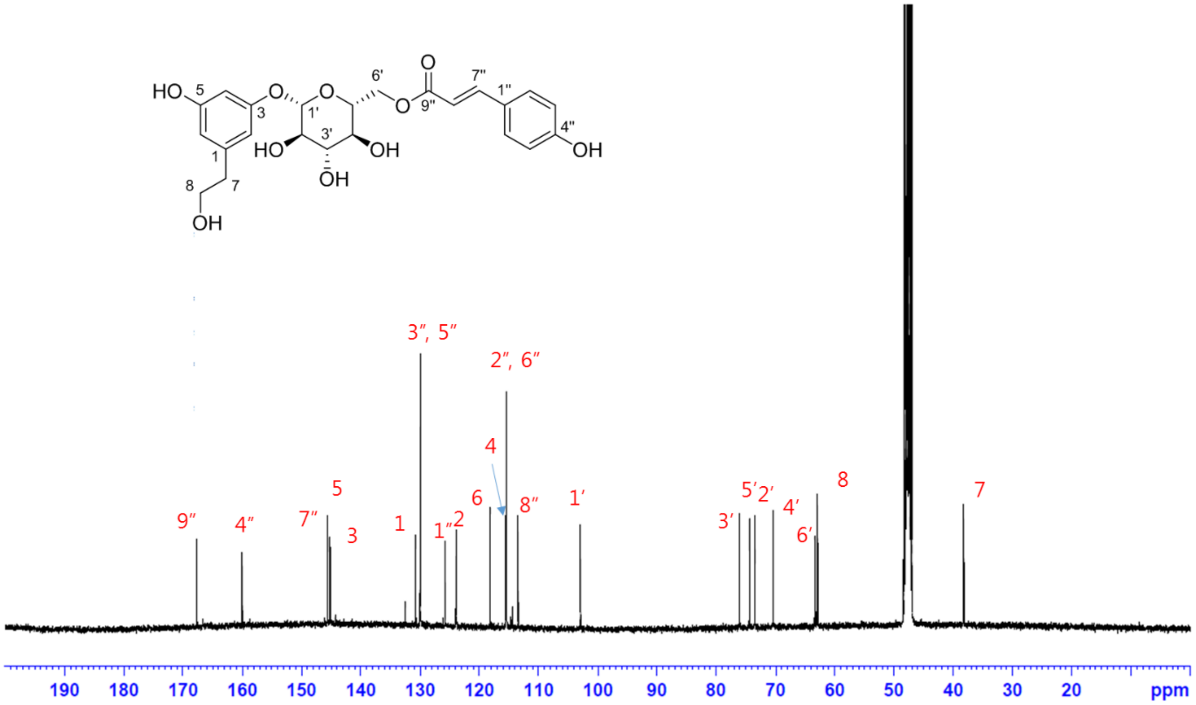

Figure S12.^{13}C-NMR spectrum of compound **3** (CD_3OD, 100 MHz)

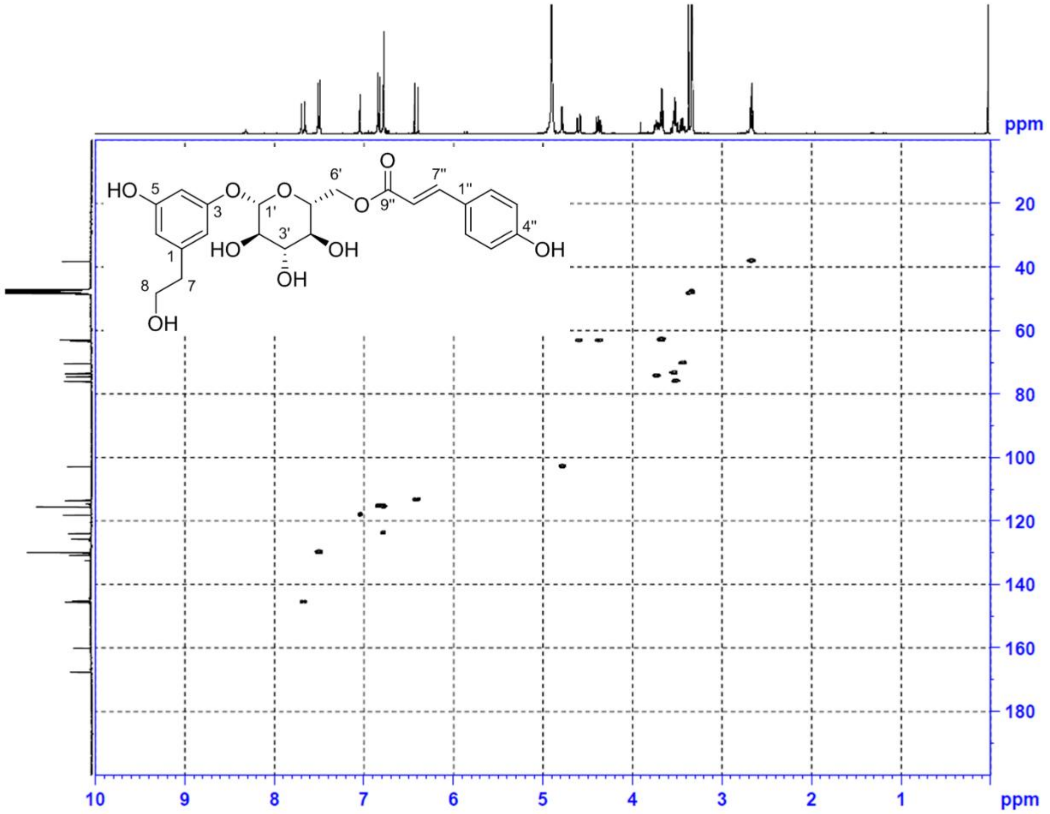

Figure S13. HSQC spectrum of compound **3** (CD_3OD, 100 MHz)

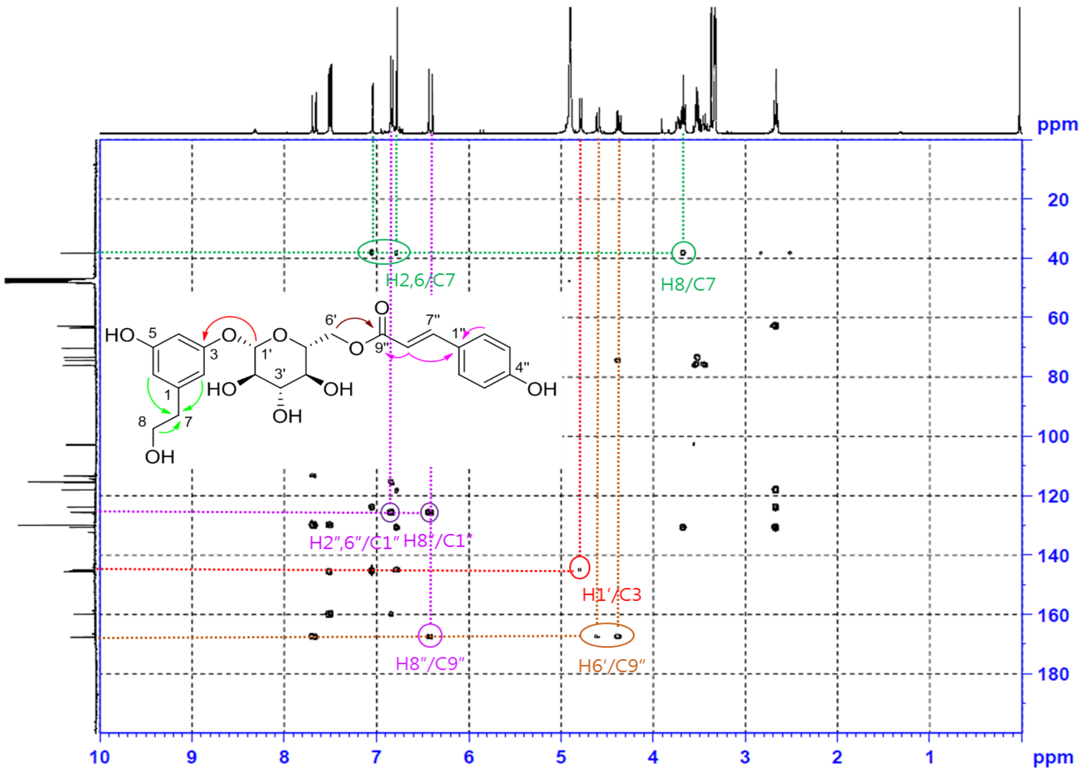

Figure S14. HMBC spectrum of compound **3** (CD_3OD, 100 MHz)

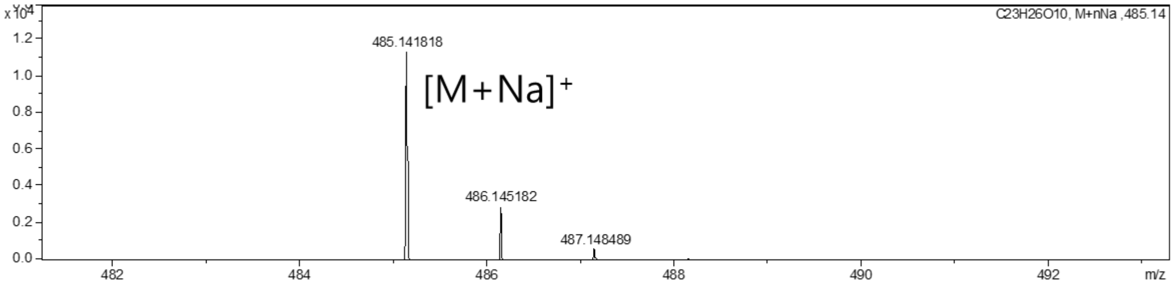

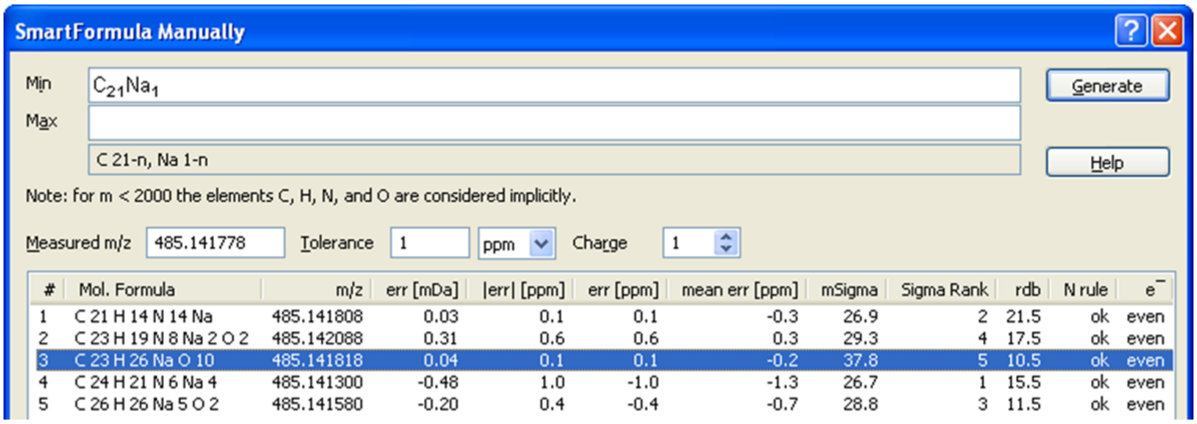

SmartFormula Manually

Min: $C_{21}Na_1$

Max:

C 21-n, Na 1-n

Generate

Help

Note: for m < 2000 the elements C, H, N, and O are considered implicitly.

Measured m/z: 485.141778 Tolerance: 1 ppm Charge: 1

#	Mol. Formula	m/z	err [mDa]	|err| [ppm]	err [ppm]	mean err [ppm]	mSigma	Sigma Rank	rdb	N rule	e⁻
1	C 21 H 14 N 14 Na	485.141808	0.03	0.1	0.1	-0.3	26.9	2	21.5	ok	even
2	C 23 H 19 N 8 Na 2 O 2	485.142088	0.31	0.6	0.6	0.3	29.3	4	17.5	ok	even
3	C 23 H 26 Na O 10	485.141818	0.04	0.1	0.1	-0.2	37.8	5	10.5	ok	even
4	C 24 H 21 N 6 Na 4	485.141300	-0.48	1.0	-1.0	-1.3	26.7	1	15.5	ok	even
5	C 26 H 26 Na 5 O 2	485.141580	-0.20	0.4	-0.4	-0.7	28.8	3	11.5	ok	even

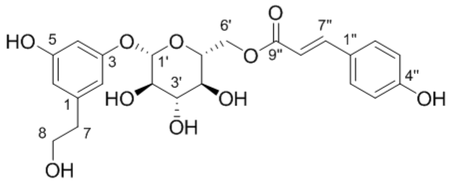

Chemical Formula: $C_{23}H_{26}O_{10}$
Exact Mass: 462.1526

HRESI-TOF-MS
m/z 485.1418
(calcd. for $C_{23}H_{26}NaO_{10}$ 485.1424)

Figure S15. HRESI-TOF-MS spectrum of compound **3**

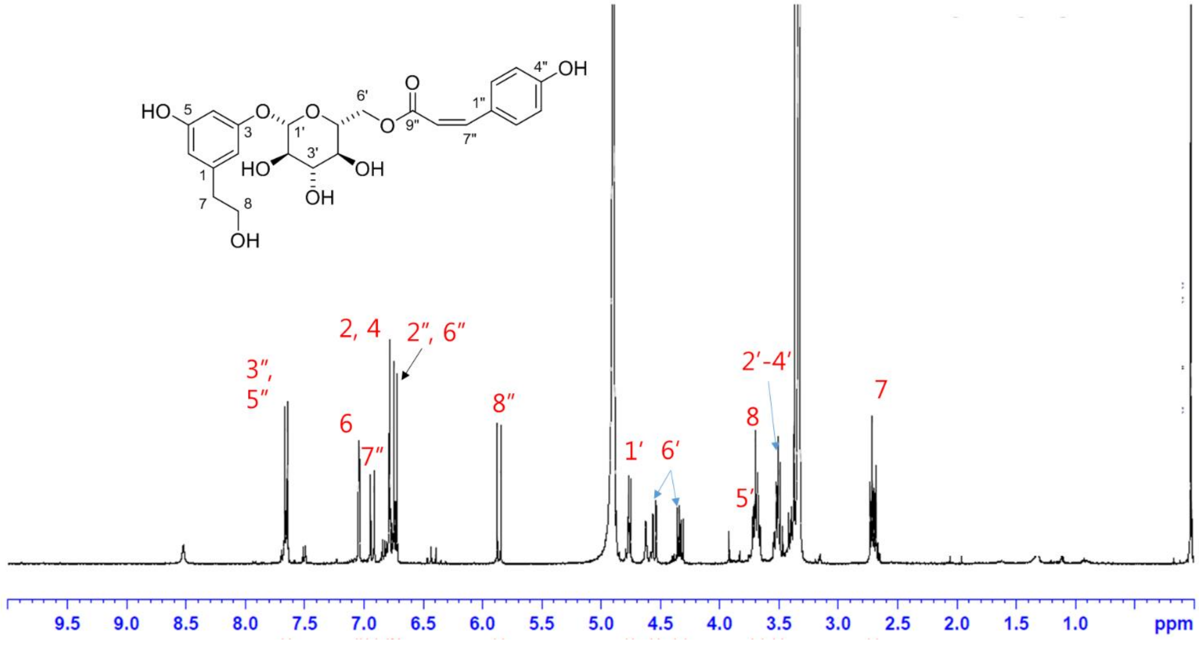

Figure S16. ^{1}H-NMR spectrum of compound **4** (CD_3OD, 400 MHz)

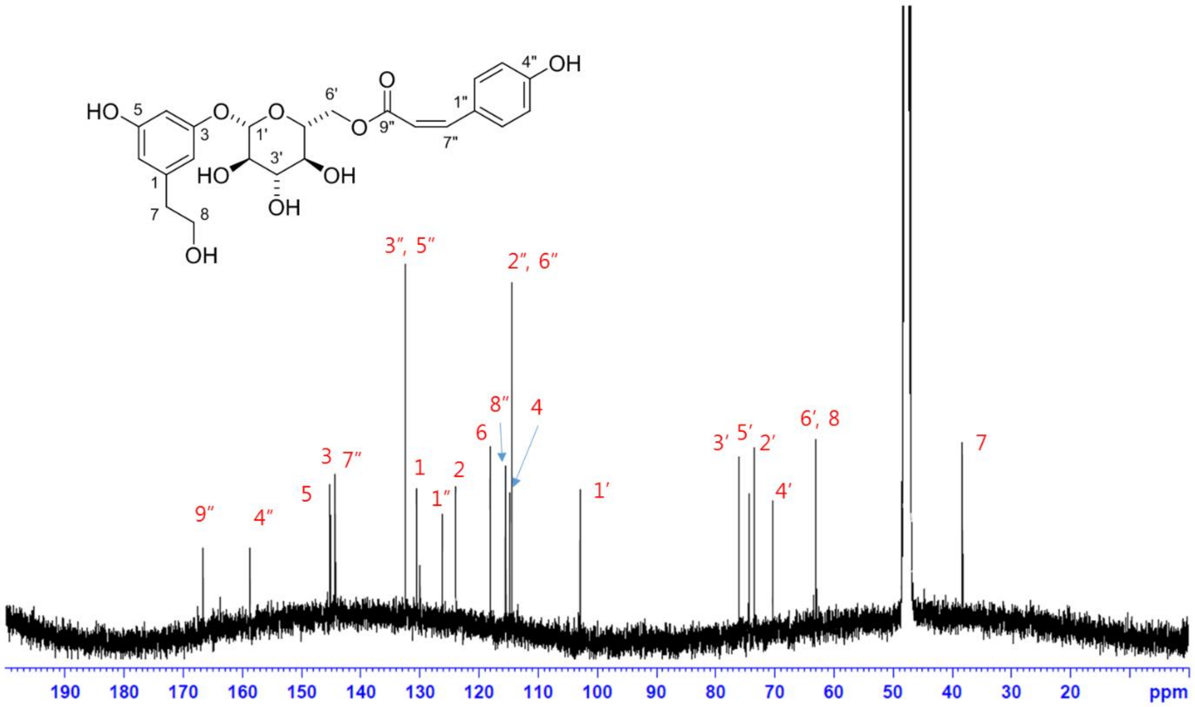

Figure S17. ^{13}C-NMR spectrum of compound **4** (CD_3OD, 100 MHz)

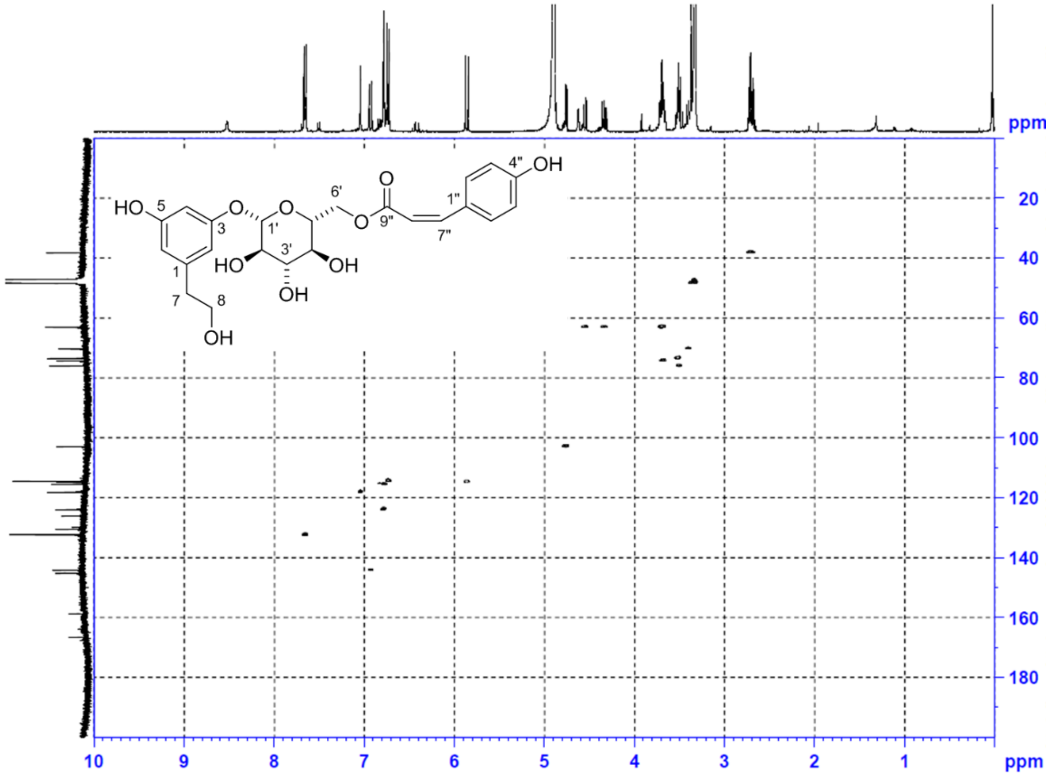

Figure S18. HSQC spectrum of compound **4** (CD_3OD, 100 MHz)

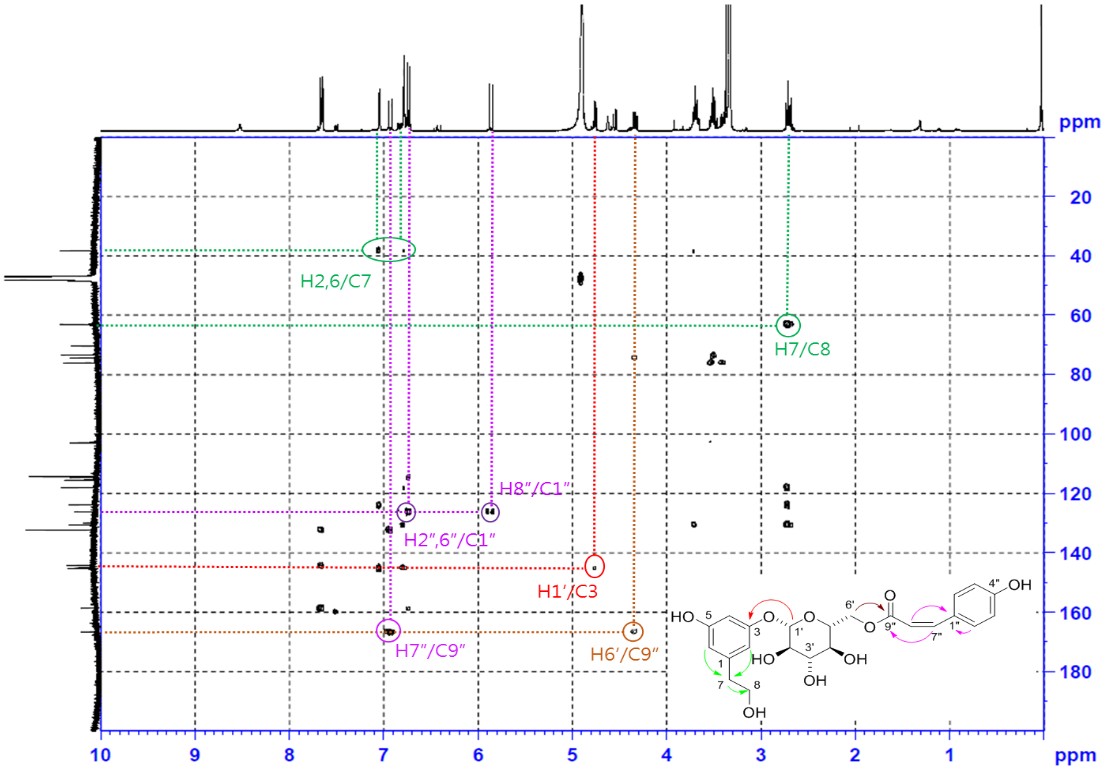

Figure S19. HMBC spectrum of compound **4** (CD_3OD, 100 MHz)

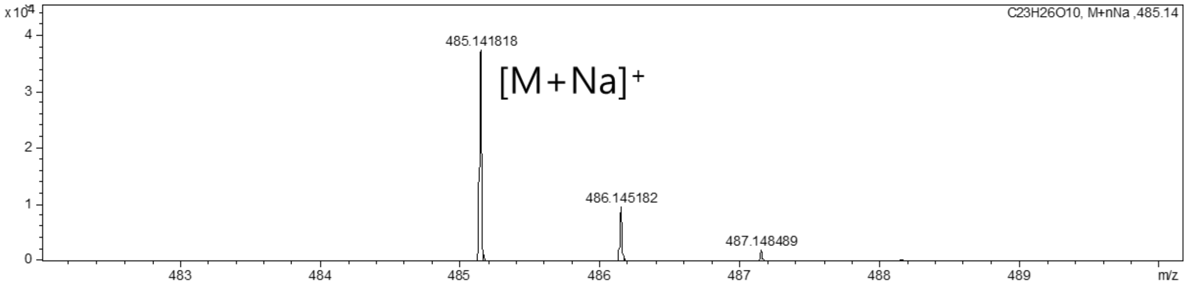

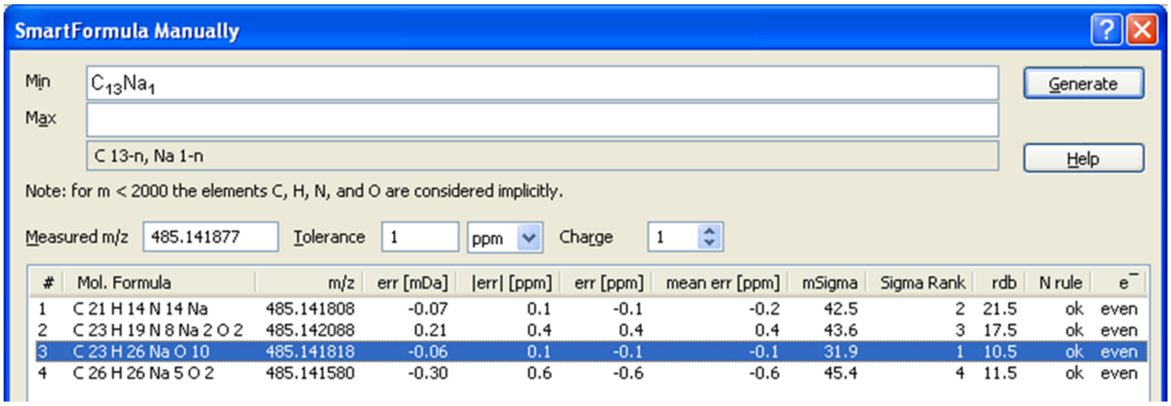

SmartFormula Manually

Min: $C_{13}Na_1$

Max:

C 13-n, Na 1-n

Note: for m < 2000 the elements C, H, N, and O are considered implicitly.

Measured m/z: 485.141877 Tolerance: 1 ppm Charge: 1

#	Mol. Formula	m/z	err [mDa]	|err| [ppm]	err [ppm]	mean err [ppm]	mSigma	Sigma Rank	rdb	N rule	e⁻
1	C 21 H 14 N 14 Na	485.141808	-0.07	0.1	-0.1	-0.2	42.5	2	21.5	ok	even
2	C 23 H 19 N 8 Na 2 O 2	485.142088	0.21	0.4	0.4	0.4	43.6	3	17.5	ok	even
3	C 23 H 26 Na O 10	485.141818	-0.06	0.1	-0.1	-0.1	31.9	1	10.5	ok	even
4	C 26 H 26 Na 5 O 2	485.141580	-0.30	0.6	-0.6	-0.6	45.4	4	11.5	ok	even

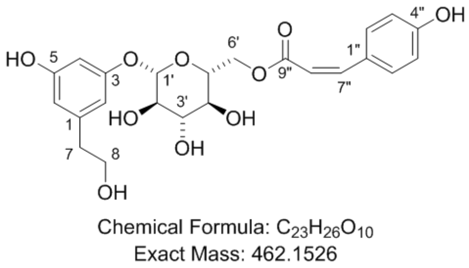

HRESI-TOF-MS
m/z 485.1418
(calcd. for $C_{23}H_{26}NaO_{10}$ 485.1424)

Figure S20. HRESI-TOF-MS spectrum of compound **4**

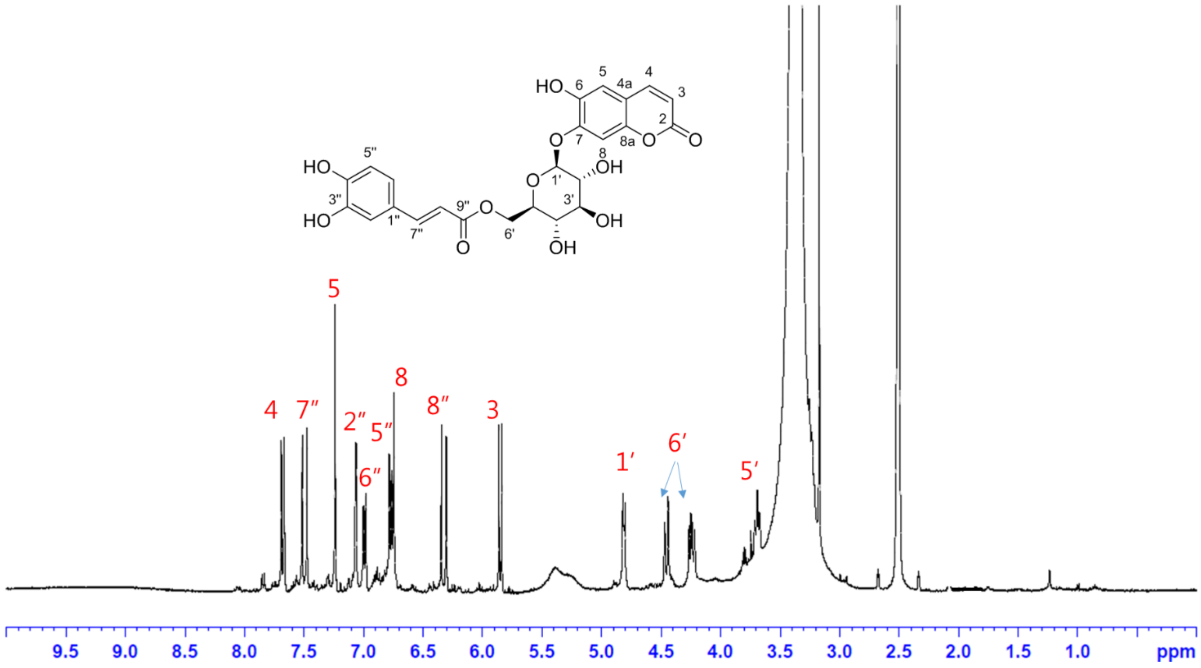

Figure S21. ^{1}H-NMR spectrum of compound **5** (DMSO-d_6, 400 MHz)

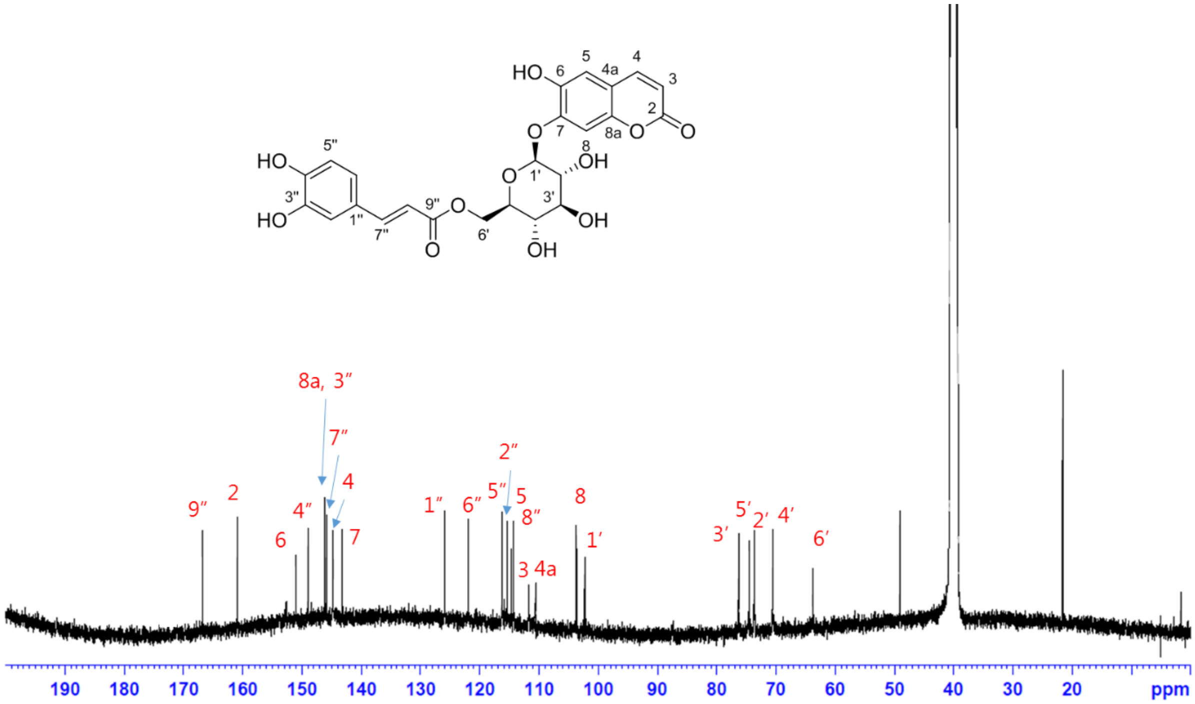

Figure S22. ^{13}C-NMR spectrum of compound **5** (DMSO-d_6, 100 MHz)

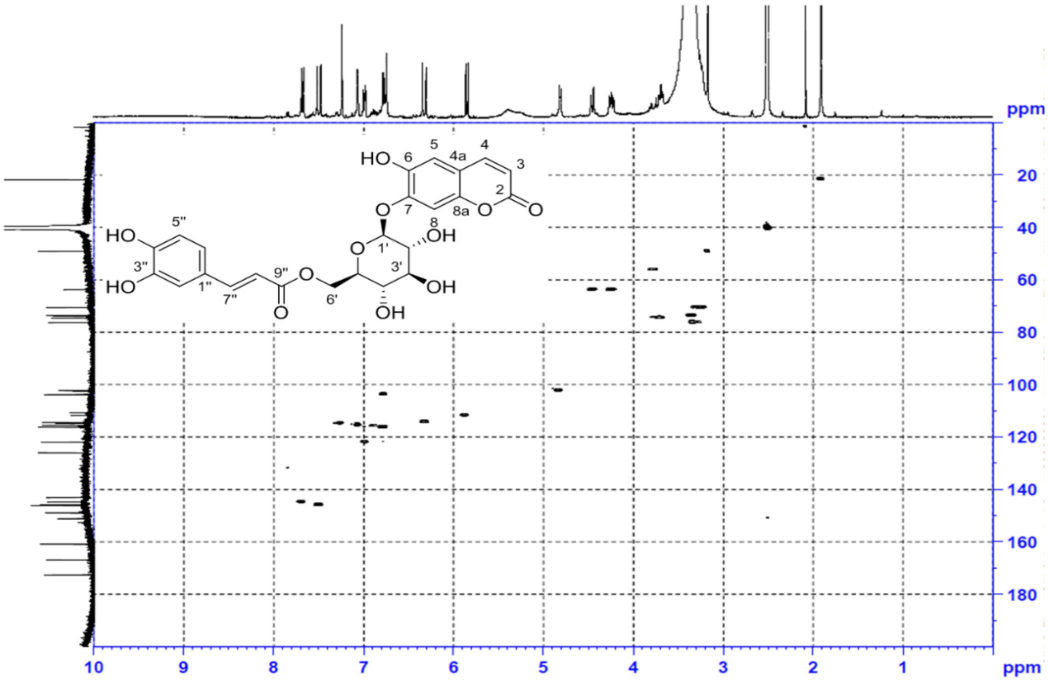

Figure S23. HSQC spectrum of compound **5** (DMSO-d_6, 100 MHz)

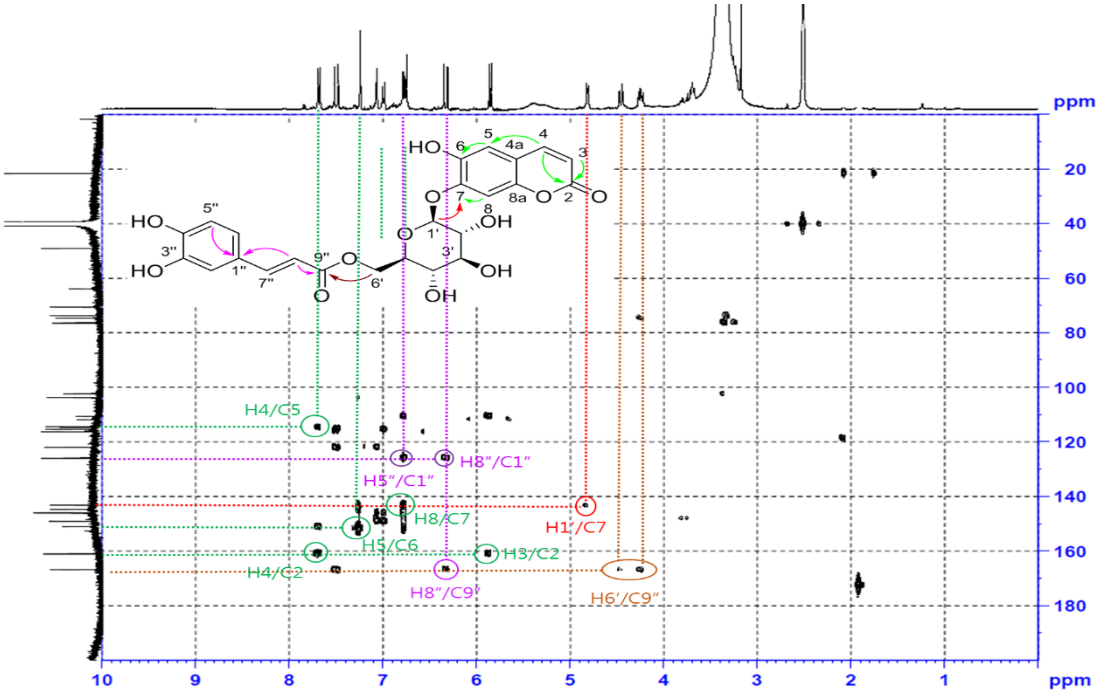

Figure S24. HMBC spectrum of compound **5** (DMSO-d_6, 100 MHz)

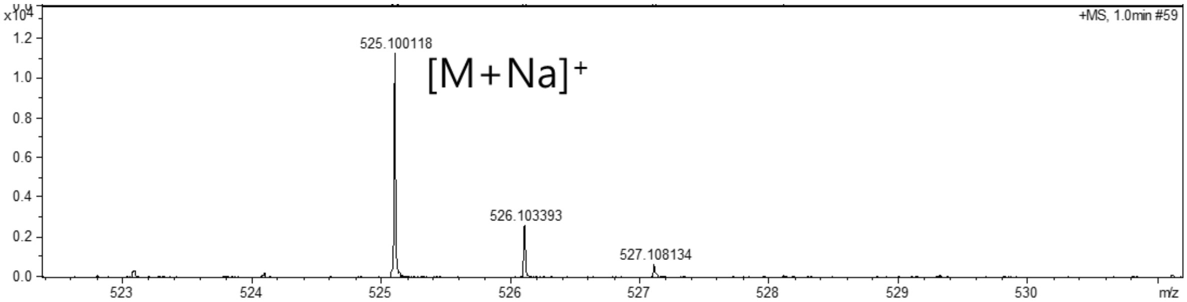

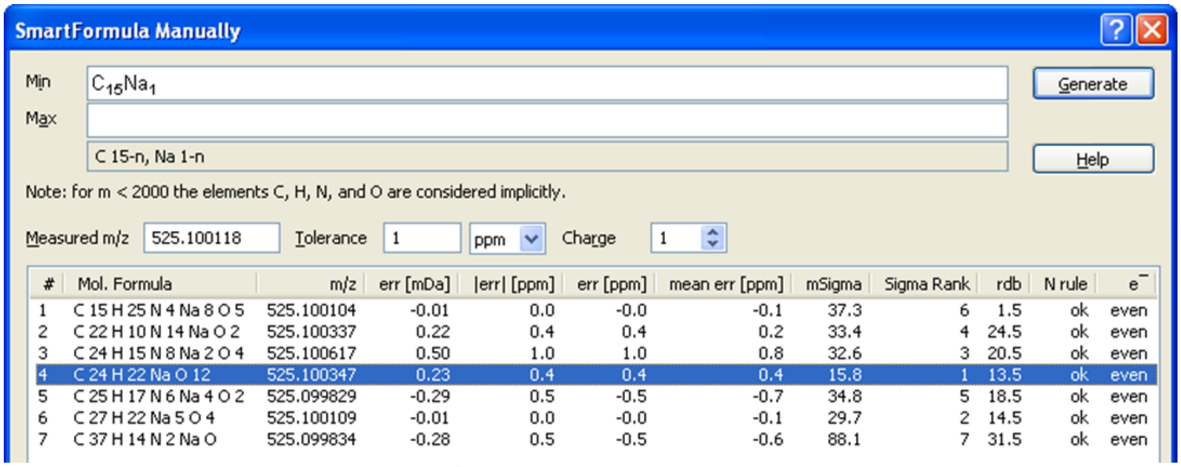

SmartFormula Manually

Min: $C_{15}Na_1$

Max:

C 15-n, Na 1-n

Generate

Help

Note: for m < 2000 the elements C, H, N, and O are considered implicitly.

Measured m/z: 525.100118 | Tolerance: 1 ppm | Charge: 1

#	Mol. Formula	m/z	err [mDa]	|err| [ppm]	err [ppm]	mean err [ppm]	mSigma	Sigma Rank	rdb	N rule	e⁻
1	C 15 H 25 N 4 Na 8 O 5	525.100104	-0.01	0.0	-0.0	-0.1	37.3	6	1.5	ok	even
2	C 22 H 10 N 14 Na O 2	525.100337	0.22	0.4	0.4	0.2	33.4	4	24.5	ok	even
3	C 24 H 15 N 8 Na 2 O 4	525.100617	0.50	1.0	1.0	0.8	32.6	3	20.5	ok	even
4	C 24 H 22 Na O 12	525.100347	0.23	0.4	0.4	0.4	15.8	1	13.5	ok	even
5	C 25 H 17 N 6 Na 4 O 2	525.099829	-0.29	0.5	-0.5	-0.7	34.8	5	18.5	ok	even
6	C 27 H 22 Na 5 O 4	525.100109	-0.01	0.0	-0.0	-0.1	29.7	2	14.5	ok	even
7	C 37 H 14 N 2 Na O	525.099834	-0.28	0.5	-0.5	-0.6	88.1	7	31.5	ok	even

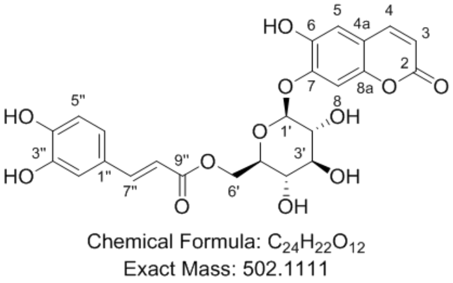

HRESI-TOF-MS
m/z 525.1003
(calcd. for $C_{24}H_{22}NaO_{12}$ 525.1009)

Figure S25. HRESI-TOF-MS spectrum of compound **5**.
